# Emergence of a New Population of *Rathayibacter toxicus*: An Ecologically Complex, Geographically Isolated Bacterium

**DOI:** 10.1371/journal.pone.0156182

**Published:** 2016-05-24

**Authors:** Mohammad Arif, Grethel Y. Busot, Rachel Mann, Brendan Rodoni, Sanzhen Liu, James P. Stack

**Affiliations:** 1 Department of Plant Pathology, Kansas State University, Manhattan, Kansas, United States of America; 2 Department of Economic Development, Jobs, Transport and Resources, Biosciences Research Division, Bundoora, Victoria, Australia; 3 Plant Biosecurity Cooperative Research Centre, Canberra, Australia; Virginia Tech, UNITED STATES

## Abstract

*Rathayibacter toxicus* is a gram-positive bacterium that infects the floral parts of several Poaceae species in Australia. Bacterial ooze is often produced on the surface of infected plants and bacterial galls are produced in place of seed. *R*. *toxicus* is a regulated plant pathogen in the U.S. yet reliable detection and diagnostic tools are lacking. To better understand this geographically-isolated plant pathogen, genetic variation as a function of geographic location, host species, and date of isolation was determined for isolates collected over a forty-year period. Discriminant analyses of recently collected and archived isolates using Multi-Locus Sequence Typing (MLST) and Inter-Simple Sequence Repeats (ISSR) identified three populations of *R*. *toxicus*; RT-I and RT-II from South Australia and RT-III from Western Australia. Population RT-I, detected in 2013 and 2014 from the Yorke Peninsula in South Australia, is a newly emerged population of *R*. *toxicus* not previously reported. Commonly used housekeeping genes failed to discriminate among the *R*. *toxicus* isolates. However, strategically selected and genome-dispersed MLST genes representing an array of cellular functions from chromosome replication, antibiotic resistance and biosynthetic pathways to bacterial acquired immunity were discriminative. Genetic variation among isolates within the RT-I population was less than the within-population variation for the previously reported RT-II and RT-III populations. The lower relative genetic variation within the RT-I population and its absence from sampling over the past 40 years suggest its recent emergence. RT-I was the dominant population on the Yorke Peninsula during the 2013–2014 sampling period perhaps indicating a competitive advantage over the previously detected RT-II population. The potential for introduction of this bacterial plant pathogen into new geographic areas provide a rationale for understanding the ecological and evolutionary trajectories of *R*. *toxicus*.

## Introduction

*Rathayibacter toxicus* is a nematode-vectored, gram-positive bacterial plant pathogen with a narrow host range (certain species in the Poaceae, e.g., *Lolium rigidum*) and a limited geographic distribution (parts of Australia and South Africa) [1, 2, 3, 4, 5]. The bacterium causes a gummosis disease on *L*. *rigidum* Gaudin (annual ryegrass), *Polypogon monspeliensis* (L.) Desf. (annual beard grass), and *Agrostis avenacea* J.F. Gmel. (bent grass or blown-grass) [1]. In addition to the plant symptoms described above, the production of a tunicamycin-like toxin by *R*. *toxicus* causes lethal toxicoses in horses and livestock that feed on infected plants [1, 3, 6, 7, 8]. Although less frequent in recent years, outbreaks of *R*. *toxicus* have been common in South Australia and Western Australia for over fifty years [3]. The decline in *R*. *toxicus* outbreaks in Australia in recent years may be due to better management of its invasive primary host, *Lolium rigidum*. The international trade of seed and hay of *L*. *rigidum* and other *R*. *toxicus* host species increases the risk of its movement to new geographic areas. The presence of the bacterium has not been reported in the U.S.; *R*. *toxicus* has only been confirmed in the southern hemisphere. *R*. *toxicus* was designated a U.S. Select Agent in 2008 as a consequence of its ability to cause disease in plants and toxicities in animals [6]. There is a phytosanitary inspection requirement for export of hay from Australia [9]. Early detection and accurate identification are prerequisite to the successful management of newly introduced species [10]. However, the accuracy of molecular-based diagnostics is dependent upon the level of understanding of genetic variation in the target population.

The genetic structure of microbial populations is determined by the capacity for gene flow, host range and variation, and geographic isolation of the microbe, host, or vector [11, 12]. Several plant species have been reported as natural hosts for *R*. *toxicus* [3, 6, 13] and multiple *Anguina* species have been reported to vector *R*. *toxicus* in a host-specific manner [14, 15]; the ecology of *R*. *toxicus* is conducive to the evolution and maintenance of genetic structure. The variation among populations of *R*. *toxicus* has been identified using isozyme analysis, amplified fragment-length polymorphisms (AFLP) and pulsed-field gel electrophoresis; *R*. *toxicus* populations from Western Australia were genetically distinct from South Australia populations [16, 17, 18, 19]. Those studies were based on *R*. *toxicus* isolates collected from 1973 to 1991 and one isolate collected in 2001. Little is known about the current population structure of *R*. *toxicus*.

Several approaches exist for achieving discrimination at high levels of taxonomic resolution (i.e., strain or population) including multi-locus sequence typing (MLST) and neutral locus-based approaches (e.g., SSR: simple sequence repeats, ISSR: inter simple sequence repeat, AFLP). ISSRs are the regions that extend between two simple sequence repeats [20]. ISSRs were effectively used to track individual strains *of Clavibacter michiganensis* subsp. *michiganesis*, closely related to *Rathayibacter*, in Southern Turkey, and offer great potential to determine genetic diversity in this group of bacteria [21]. MLST is a widely used strategy for discriminating among bacterial strains in epidemiological and evolutionary studies [22, 23, 24, 25]. Most applications of MLST rely on partial gene sequencing of commonly accepted conserved genes (often referred to as “house-keeping” genes) [24, 26].

The purpose of this study was to genetically analyze *R*. *toxicus* isolates collected over a forty year period from two geographic areas in Australia using a strategically designed six-gene MLST approach and ISSR analyses. Understanding the population structure of this high consequence bacterial pathogen would be helpful to understanding the epidemiology of the disease; it will also facilitate the development of accurate, robust diagnostic tools and effective disease management practices, as well as support better biosecurity decisions.

## Materials and Methods

### Ethics Statement

As all handling of *R*. *toxicus*-infected plants and live cultures of *R*. *toxicus*, and other *Rathayibacter* species, was conducted in Australia, specific permissions from government agencies or regulatory bodies were not required for the collection or processing of the plant materials used in this study. Endangered or protected species were not collected or used in this study. No samples were collected from endangered or protected field sites.

### Sample Collection, Pathogen Isolation and DNA Purification

**Fig 1:**
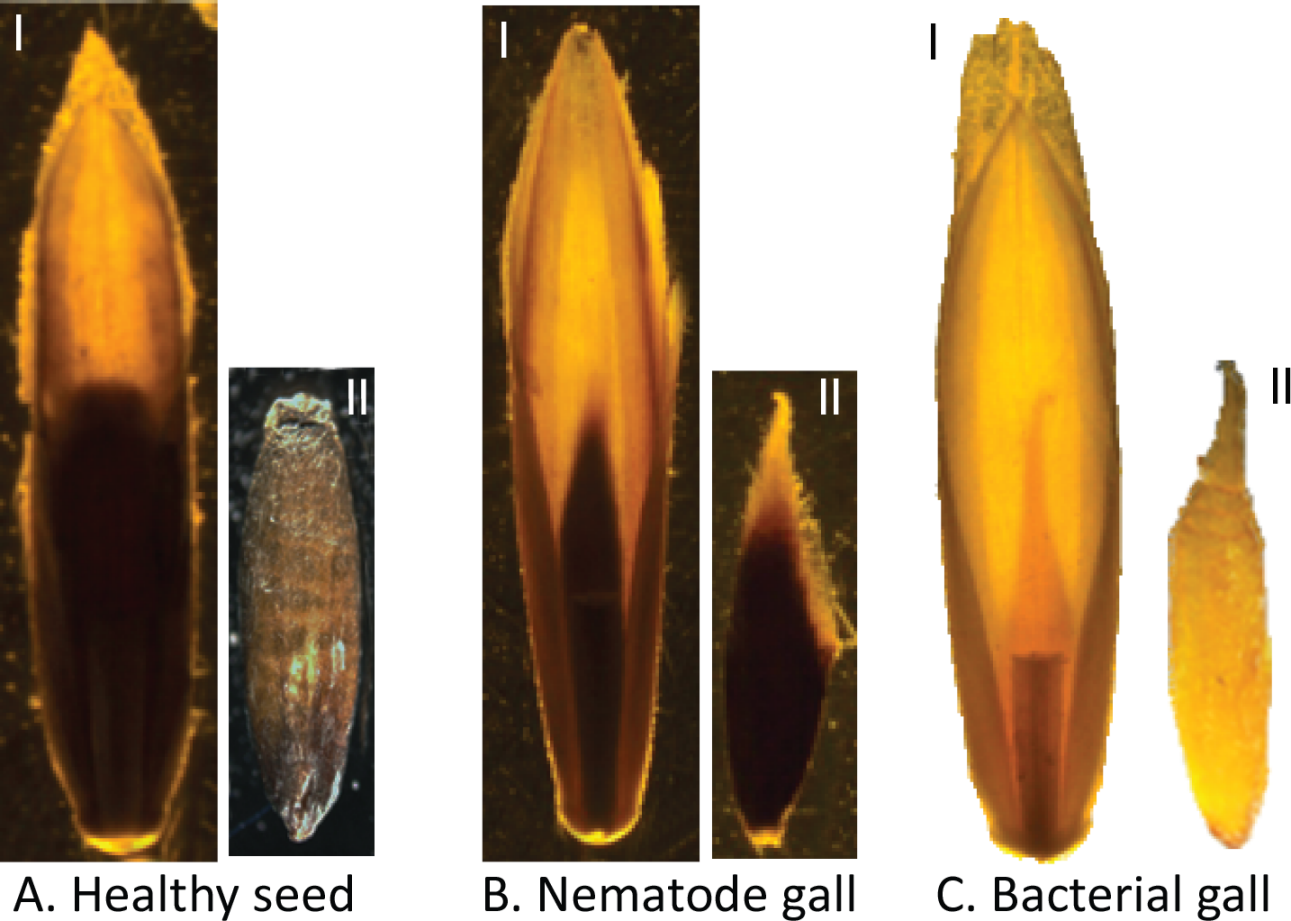
Annual ryegrass (*Lolium rigidum*) florets with a seed, a nematode gall, or a bacterial gall inside. (A) i) healthy seed inside floret, ii) healthy seed isolated from floret; (B) i) nematode gall (pointed and black in color) inside floret in place of seed, ii) nematode gall isolated from floret; and (C) i) bacterial gall (pointed and bright yellow in color) inside floret in place of seed, ii) bacterial gall isolated from floret.

To obtain current isolates of *R*. *toxicus*, field collections were conducted in spring 2013 and summer 2014 in Western Australia and South Australia where *R*. *toxicus* is indigenous. Annual ryegrass (*L*. *rigidum*) samples were collected over a wide geographic area in both states (S1 Table). In the laboratory, bacterial galls were visually identified by observation of plant samples over a fluorescent light box (Fig 1). Bacterial galls were surface sterilized using 70% ethanol for 45 sec followed by two washes of sterile water, each for 30 sec. Each surface sterilized bacterial gall was cut into small pieces (5–8) and plated onto 523M agar [6] medium in a biosafety cabinet. After 6–10 days at 26°C, bacterial growth from small pieces of gall was streaked onto a 523M medium plate to obtain single colonies. After 6–10 days growth, single colonies were streaked onto 523M medium to obtain single colony cultures. A total of 39 isolates of *R*. *toxicus* was obtained (Table 1). Isolates of *R*. *toxicus*, *Rathayibacter tritici* and *Rathayibacter agropyri* were sourced from the South Australia Research and Development Institute (SARDI; Table 1) in Adelaide, Australia. Isolates of *Rathayibacter iranicus* and *Rathayibacter rathayi* were sourced from International Collection of Microorganism from Plants (ICMP; Table 1). Purified DNA of isolates FH81, FH83, FH85, FH87, FH100, FH138, FH141 and FH147 [16] were sourced from the University of Nebraska, Lincoln. All isolates used in this study were grown on 523M agar medium plates. Genomic DNA was extracted from cultures using the Qiagen DNeasy Blood & Tissue Kit (Qiagen, Valencia, CA), and Blood & Cell Culture DNA Midi Kit (Qiagen) according to the manufacturer’s instructions. Isolated genomic DNA concentrations were calculated using a NanoDrop 2000c spectrophotometer (Thermo Fisher Scientific Inc., Worcester, MA).

**Table 1:** Isolates recently collected in 2013–14 from South Australia and old isolates received from different culture collections, universities and institutes were used to determine the population structure of *Rathayibacter toxicus*.

No	Genus/Species	Isolate Code	Other names associated	Population Type	Host	Year of Collection	Location/Source
1	*Rathayibacter toxicus*	SA03-02	-	RT-I	ARG	2014	Corny Point, SA
2	*R*. *toxicus*	SA03-03	-	RT-I	ARG	2014	Corny Point, SA
3	*R*. *toxicus*	SA03-04	-	RT-I	ARG	2014	Corny Point, SA
4	*R*. *toxicus*	SA03-08	-	RT-I	ARG	2014	Corny Point, SA
5	*R*. *toxicus*	SA03-14	-	RT-I	ARG	2014	Corny Point, SA
6	*R*. *toxicus*	SA03-15	-	RT-I	ARG	2014	Corny Point, SA
7	*R*. *toxicus*	SA03-16	-	RT-I	ARG	2014	Corny Point, SA
8	*R*. *toxicus*	SA03-17	-	RT-I	ARG	2014	Corny Point, SA
9	*R*. *toxicus*	SA03-18	-	RT-I	ARG	2014	Corny Point, SA
10	*R*. *toxicus*	SA03-19	-	RT-I	ARG	2014	Corny Point, SA
11	*R*. *toxicus*	SA03-20	-	RT-I	ARG	2014	Corny Point, SA
12	*R*. *toxicus*	SA03-21	-	RT-I	ARG	2014	Corny Point, SA
13	*R*. *toxicus*	SA03-22	-	RT-I	ARG	2014	Corny Point, SA
14	*R*. *toxicus*	SA03-23	-	RT-I	ARG	2014	Corny Point, SA
15	*R*. *toxicus*	SA03-24	-	RT-I	ARG	2014	Corny Point, SA
16	*R*. *toxicus*	SA03-25	-	RT-I	ARG	2014	Corny Point, SA
17	*R*. *toxicus*	SA03-26	-	RT-I	ARG	2014	Corny Point, SA
18	*R*. *toxicus*	SA03-27	-	RT-I	ARG	2014	Corny Point, SA
19	*R*. *toxicus*	SA03-28	-	RT-I	ARG	2014	Corny Point, SA
20	*R*. *toxicus*	SA08-03	-	RT-I	ARG	2014	Lake Sunday, SA
21	*R*. *toxicus*	SA08-07	-	RT-II	ARG	2014	Lake Sunday, SA
22	*R*. *toxicus*	SA08-08	-	RT-I	ARG	2014	Lake Sunday, SA
23	*R*. *toxicus*	SA08-09	-	RT-I	ARG	2014	Lake Sunday, SA
24	*R*. *toxicus*	SA08-11	-	RT-I	ARG	2014	Lake Sunday, SA
25	*R*. *toxicus*	SA08-13	-	RT-I	ARG	2014	Lake Sunday, SA
26	*R*. *toxicus*	SA08-16	-	RT-I	ARG	2014	Lake Sunday, SA
27	*R*. *toxicus*	SA19-02	-	RT-I	ARG	2013	Yorketown, SA
28	*R*. *toxicus*	SA19-03	-	RT-II	ARG	2013	Yorketown, SA
29	*R*. *toxicus*	SA19-04	-	RT-I	ARG	2013	Yorketown, SA
30	*R*. *toxicus*	SA19-05	-	RT-II	ARG	2013	Yorketown, SA
31	*R*. *toxicus*	SA19-06	-	RT-I	ARG	2013	Yorketown, SA
32	*R*. *toxicus*	SA19-07	-	RT-I	ARG	2013	Yorketown, SA
33	*R*. *toxicus*	SA19-08	-	RT-II	ARG	2013	Yorketown, SA
34	*R*. *toxicus*	SA19-09	-	RT-I	ARG	2013	Yorketown, SA
35	*R*. *toxicus*	SA19-10	-	RT-II	ARG	2013	Yorketown, SA
36	*R*. *toxicus*	SA19-11	-	RT-I	ARG	2013	Yorketown, SA
37	*R*. *toxicus*	SA19-12	-	RT-I	ARG	2013	Yorketown, SA
38	*R*. *toxicus*	SA19-13	-	RT-I	ARG	2013	Yorketown, SA
39	*R*. *toxicus*	SA19-14	-	RT-II	ARG	2013	Yorketown, SA
40	*R*. *toxicus*	SAC3368	WAC3368, WSM185	RT-II	ARG	1981	SA
41	*R*. *toxicus*	SAC3387	WAC3387, SARG2A	RT-II	ARG	1981	SA
42	*R*. *toxicus*	SAC7056	WAC7056, CS14, ICMP 9525, JCM9669, NCPPB 3552, D84127	RT-II	ARG	1983	Murray Bridge, SA
43	*R*. *toxicus*	WAC3371	WSM189	RT-III	LCG	1978	Gnowangerup, WA
44	*R*. *toxicus*	WAC3372	WSM190	RT-III	BO	1978	Gnowangerup, WA
45	*R*. *toxicus*	WAC3373	WSM194	RT-III	PG	1978	Gnowangerup, WA
46	*R*. *toxicus*	WAC3396	WSM447, CS30	RT-III	Oat	1980	Gnowangerup, WA
47	*R*. toxicus*	FH100	SE3	RT-II	ABS	1991	Southeastern, SA
48	*R*. toxicus*	FH83	CRS2 dark	RT-II	ARG	1975	SA
49	*R*. toxicus*	FH85	CRS3	RT-II	ARG	1975	SA
50	*R*. toxicus*	FH147	CS33, ICMP 9526	RT-II	ARG	1984	SA
51	*R*. toxicus*	FH141	CS2	II	ARG	1983	SA
52	*R*. toxicus*	FH81	CRK73dy dark	III	ARG	1973	WA
53	*R*. toxicus*	FH138	CS28, ICMP 6307	RT-III	ARG	1978	WA
54	*R*. toxicus*	FH87	CRW1 light	RT-III	ARG	1974	WA
55	*R*. *tritici*	WAC7055	CS103	-	Wheat	1991	Carnamah, WA
56	*R*. *tritici*	WAC9601	CS21, NCPPB 1857, ICPBCT102, ATCC11403	-	RG	-	South Perth, WA
57	*R*. *tritici*	WAC9602	CS101	-	RG	-	South Perth, WA
58	*R*. *agropyri*	WAC9620	CS106, 52-4-4	-	RG	-	South Perth, WA
59	*R*. *agropyri*	WAC9594	CS35, 41-9-5, CA-1	-	RG	-	South Perth, WA
60	*R*. *iranicus*	ICMP 12831	-	-	Wheat	1994	Iran
61	*R*. *iranicus*	ICMP 13126	-	-	Wheat	1994	Iran
62	*R*. *iranicus*	ICMP 13127	-	-	Wheat	1994	Iran
63	*R*. *iranicus*	ICMP 3496	-	-	Wheat	1996	Iran
64	*R*. *rathayi*	ICMP 2579	-	-	DG	-	United Kingdom
65	*R*. *rathayi*	ICMP 2574	-	-	DG	1968	New Zealand
66	*R*. *rathayi*^#^	WAC3369	-	-	ARG	-	WA
67	*Clavibacter michiganensis* subsp. nebraskensis**	NCPPB 2581			-	-	NCBI GenBank
68	*R*. caricis**	VKM Ac-1799	-	-	-	-	NCBI GenBank
69	*R*. festucae**	DSM 15932	-	-	-	-	NCBI GenBank
70	*R*. festucae**	UCM Ac619	-	-	-	-	NCBI GenBank

*Only DNA was available for this study

**DNA sequences for these *Rathayibacter* species and *Clavibacter michiganensis* subsp. *nebraskensis* were retrieved from NCBI GenBank

^#^This isolate was received from a culture collection as *R*. *toxicus* but we identified it as *R*. *rathayi* based on 16S ribosomal sequences; ABS-annual beard grass (*Polypogon monspeliensis*); ARG-annual ryegrass (*Lolium rigidum*); RG-ryegrass (*Lolium* sp.); LCG-lesser canary grass (*Phalaris minor*); PG-paradoxa grass (*Phalaris paradoxa*); BO-black oat (*Avena fatua*); DG- *Dactylis glomerata* L. Oat (*Avena sativa*); wheat (*Triticum* sp./*Triticum aestivum*). SA-South Australia, Australia; WA-Western Australia, Australia; All the isolates collected during 2013–14 are from this study.

### Gene Selection, Primer Design and Gene-Specific PCRs

A total of 7 genes including, 16S ribosomal RNA gene, chromosome partition protein SMC, tRNA dihydrouridine synthase, cysteine desulfurase, CRISPR-associated protein *cse4*, vancomycin-resistance protein, and *secA* ATPase were selected for multi-locus sequence typing (MLST) analysis. The whole genome sequence of *R*. *toxicus* (accession number ASM42532v1) was retrieved from the NCBI GenBank database (http://www.ncbi.nlm.nih.gov/) and used for primer design of targeted genes (Table 2) of *R*. *toxicus*. Primers were designed using Geneious® 7.1.7 and online software Primer3 following the protocol of Arif and Ochoa-Corona [27]. Isolate identity was verified as *R*. *toxicus* based on 16S ribosomal RNA gene sequence homology; PCR primers R16sF1 (5’-TAACACGTGAGTAACCTGCC-3’) and R16sR1 (5’-CATTGTAGCATGCGTGAAG-3’) were developed and used to amplify a 1110 base pair (bp) fragment of the 16S rDNA gene. Primers were synthesized by IDT (Integrated DNA Technologies, Inc., Coralville, IA).

**Table 2:** Primers designed and used to amplify MLST genes for *Rathayibacter toxicus*.

Primer Name	Primer Sequence (5’-3’)	Amplicon Size (bp)	Edited Sequence Size (bp)	PCR Conditions	Target Gene
Chr-F1	AAATGCACGTCATCGTCGGT	1014	912	T_a_ = 59°C (60 s);T_e_ = 72°C (80 s)	Chromosome partition protein SMC
Chr-R1	GTTCCCGGGCGTGCAACT				
tRNA-F1	GATGACGACGGACTGAAGGAT	1155	998	T_a_ = 59°C (60 s);T_e_ = 72°C (80 s)	tRNA dihydrouridine synthase
tRNA-R1	AACCTCGCGGGAGTCGAG				
Cys-F1	CGTGATCCGCTAATTGTCGA	1137	766	T_a_ = 55°C (60 s); T_e_ = 72°C (120 s)	Cystein desulfurase
Cys-R1	GCGACCAGAAACCCGTAG				
Crispr-F	ATGTATGTCGATATTGATATATTGCAGACCG	1110	941	T_a_ = 56°C (60 s); T_e_ = 72°C (120 s)	CRISPR-associated protein, *cse4* family
Crispr-R	TCATGAGACGCCACCGAGG				
VanA-F	ATGAACACACTGACCGTAG	1041	831	T_a_ = 52°C (60 s); T_e_ = 72°C (120 s)	Vancomycin resistant protein *vanA*
VanA-R	TCATGCCACTGTCTCCG				
SecA-F	GTGGCCTCAGTTCTCGAAAAGGTCC	1262	734*	T_a_ = 61°C (60 s); T_e_ = 72°C (120 s)	*secA* ATPase
SecA-R	ACGACCTGCTCGAACTTGACCTG				
SecA-R1	AGCGGAGTGTTGACCGACTC				

*For sequencing, secA-R1 was used instead of secA-R; T_a_-Annealing temperature; T_e_-Extension temperature

Gene-specific PCR amplifications were carried out in 25 μl of reaction mixtures containing 12.5 μl of GoTaq® G2 Hot Start Green Master Mix (Promega, Madison, WI), 0.2 μM each of the forward and reverse primers, 1 μl of DNA template and molecular grade nuclease free water (G-Biosciences, St. Louis, MO) to volume (Table 3). For the 16S ribosomal RNA partial gene amplification, the annealing and extension conditions were 56°C for 20 sec, and 72°C for 60 sec, respectively. Amplified PCR products (5 μl) were electrophoresed and separated in a 1.5% agarose gel in 1X Tris-acetate-EDTA (TAE) or Tris-borate-EDTA (TBE) buffer to confirm the specific amplification. Amplicon sizes were estimated using HyperLadder 50 bp (Bioline USA Inc., Taunton, MA). PCR amplifications were performed in a PTC-200 Peltier thermal cycler (MJ Research Inc., Watertown, MS) and DNA Engine (Biorad, Hercules, CA).

**Table 3:** Primer sequences and results for ISSR analysis of 54 isolates of *Rathayibacter toxicus* collected from South and Western Australia.

Primer Code	Primer Sequence (5’- 3’)	T_a_	Total loci	P-loci	P-loci (%)	M-loci	M-loci (%)	U-loci
P7	GTGGTGGTGGTGGTG	53°C	8	6	75	2	25	-
P14	AGACAGACAGACAGAC	48°C	10	7	70	3	30	-
P15	CAGCAGCAGCAGCAG	55°C	9	5	56	4	44	-
P16	AGCAGCAGCAGCAGC	56.5°C	5	1	20	4	80	-
UBC 807	AGAGAGAGAGAGAGAGT	48°C	9	5	56	4	44	1 (SAC3387)
UBC 810	GAGAGAGAGAGAGAGAT	48°C	15	15	100	-	-	2 (SA03-20, SA19-03)
UBC 840	GAGAGAGAGAGAGAGAYT	48°C	17	14	82	3	18	-
UBC 881	GGGTGGGGTGGGGTG	55°C	3	2	67	1	33	-
UBC 885	BHBGAGAGAGAGAGAGA	48°C	10	5	50	5	50	-
UBC 991	HVHTGTGTGTGTGTGTG	51°C	8	5	63	3	38	1 (SA03-08)
	Total		94	65		29		4
	Average			6.5	69.15	2.9	30.85	

Abbreviation for mixed base positions: Y (C, T), B (C, G, T), H (A, C, T), V (A, C, G); T_a_ = Annealing temperature; P-loci = Polymorphic loci (loci present in >1 isolate); M-loci = Monomorphic loci (loci present in all isolate); U-loci = Unique loci (loci present only in one isolate)

### Sequencing

PCR products (20 μl) were purified using the NucleoSpin Gel and PCR Clean-up kit (Macherey-Nagel Inc., Bethlehem, PA) according to the manufacturer’s instructions. Purified amplicons were quantified using a NanoDrop 2000c spectrophotometer. A total of 20 μl diluted purified DNA (2 ng/μl) was sent to Genewiz Inc., Newbury Park, CA, for direct sequencing of both strands using the specific forward and reverse primers designed for each target gene. Partial 16S ribosomal gene sequences of *Rathayibacter caricis*, *Rathayibacter festucae* and *Clavibacter michiganensis* subsp. *nebraskensis* were retrieved from NCBI nucleotide database (S1 Table).

### Inter-Simple Sequence Repeats (ISSR; Inter-Microsatellite)

ISSR amplification was performed with 10 primers (15-18mers; Table 3) [28] following the protocol of Arif et al. [29]; protocol was modified for PCR components and conditions to improve PCR amplification. Primers were synthesized by IDT (Integrated DNA Technologies, Inc.). ISSR amplification reactions were carried out in 25 μl mixtures containing 2.5 μl 10X buffer, 2.5 μl MgCl_2_ (25 mM), 2.5 μl dNTPs (2.5 mM each), 2.0 μl primer (5 μM), 0.3 μl TaKaRa Taq (5U/μl; Clontech Laboratories, Inc., Mountain View, CA) and 1 μl of DNA template (5 ng/μl), and 14.2 μl molecular grade nuclease-free water. The PCR cycling parameters were: initial denaturation at 95°C for 3 min followed by 35 cycles of denaturation at 95°C for 20 s, annealing at 48–56.5°C (Table 3) for 40 s, extension at 72°C for 2 min, and a final extension at 72°C for 5 min. A volume of 15 μl PCR product was electrophoresed in a 1.8% agarose gel in 1X TAE buffer. Amplicon sizes were estimated using HyperLadder 50 bp and 1kb ladder (Invitrogen, Carlsbad, CA). PCR amplifications were performed in a PTC-200 Peltier thermal cycler and DNA Engine.

### Data Analyses

ISSR agarose gels were manually scored and the results expressed in a binary matrix as the presence (1) or absence (0) of ISSR loci. Pairwise Jaccard’s coefficients [30] were calculated for all *R*. *toxicus* isolates based on the 94 ISSR loci using the SimQual program of NTSYSpc (version 2.21q; Exeter Biological Software, Setauket, NY). Genetic relationships among the 54 isolates of *R*. *toxicus* were calculated using the Unweighted Pair Group Method with Arithmetic Mean (UPGMA) analysis in SAHN module and neighbor-joining (NJ) analysis in Njoin module of NTSYSpc. Bootstrap resampling method in Resample module of NTSYSpc was used to generate the consensus tree with 1000 replicates. The total number of loci, the percentage polymorphism, the number of monomorphic loci, and the number of polymorphic loci were also calculated. Two-way Mantel test [31] was performed using the MxComp module of NTSYSpc 2.21q to calculate the co-phenetic correlation (r) between the two symmetric dissimilar matrices, and plotted one matrix against the other, element by element (one is cophenetic (ultrametric) obtained from COPH program and the other matrix which was used to form the cluster). Cophenetic correlation was used to measure goodness-of-fit for a cluster analysis. Principal coordinate analysis (PCOORDA; multidimensional scaling) was performed using DCENTER and EIGEN modules of NTSYSpc 2.21q to highlight the resolving power of the ordination. PCOORDA was calculated using the double-centered distance matrices (standardized by variables ‘raws’) to obtain three-dimensional (3-D) and two-dimensional (2-D) graphics. PCOORDA can be assumed as a computational alternative to principal component analysis (PCA).

AMOVA [32] was performed to examine population genetic structure of *R*. *toxicus* using GenAlEx 6.5 [33, 34]. PhiPT (Φ*pt*), an analogue of F*st*, was also calculated to describe genetic differentiation between the populations. Probability (P) for Φ*pt* was based on 999 permutations across the full data set. The Nei’s calculation of pairwise binary genetic distances (estimate of genetic difference among the populations) using ISSR data was also performed [35]. Genetic diversity was calculated (GenAlEx 6.5) for each locus using the parameters: number of different loci (Na), and the number of effective loci (Ne).

Partial gene sequences of the six genes used for MLST and the 16S ribosomal RNA gene were edited for accuracy, aligned and trees were constructed using the Geneious Tree Builder module of Geneious 7.1.7. Sequences from the six MLST genes were concatenated to generate a combined tree using NJ and UPGMA tree building methods [36, 37]. Tumura-Nei genetic distance model [38] was used to estimate branch lengths and Bootstrap resampling method (resampling with replacement) [39] was used to generate the consensus tree with 1000 replicates.

### Nucleotide Sequence Accession Numbers

The sequences for the 16S ribosomal and MLST genes analyzed in this study are available at NCBI GenBank under the following accession numbers: KT754155, KT754158-KT754159, KT760408-KT760471 (16S ribosomal RNA gene), KT875413-KT875466 (CRISPR-associated protein *cse4*), KT875521-KT875574 (chromosome partition protein gene SMC), KT875575-KT875628 (tRNA dihydrouridine synthase), KT875467-KT875520 (cysteine desulfurase), KT875683-KT875736 (vancomycin resistant protein *vanA*), and KT875629-KT875682 (*secA* ATPase). S1 Table contain accession numbers correspond to each isolate and gene.

## Results

### Sample Collection and Isolation

A total of 54 isolates of *R*. *toxicus* were used in this study, 39 isolates from plant materials collected in 2013 and 2014 from South Australia and 15 isolates from archive collections (Table 1). Surveys of AGRT-prone regions of Western Australia were unsuccessful in obtaining current isolates of *R*. toxicus. All isolates that were ultimately identified as *R*. *toxicus* were similar in growth characteristics and colony appearance, yielding dark yellow colonies after 10–14 days on 523M agar medium.

### Identity of *R*. *toxicus* Isolates

**Fig 2:**
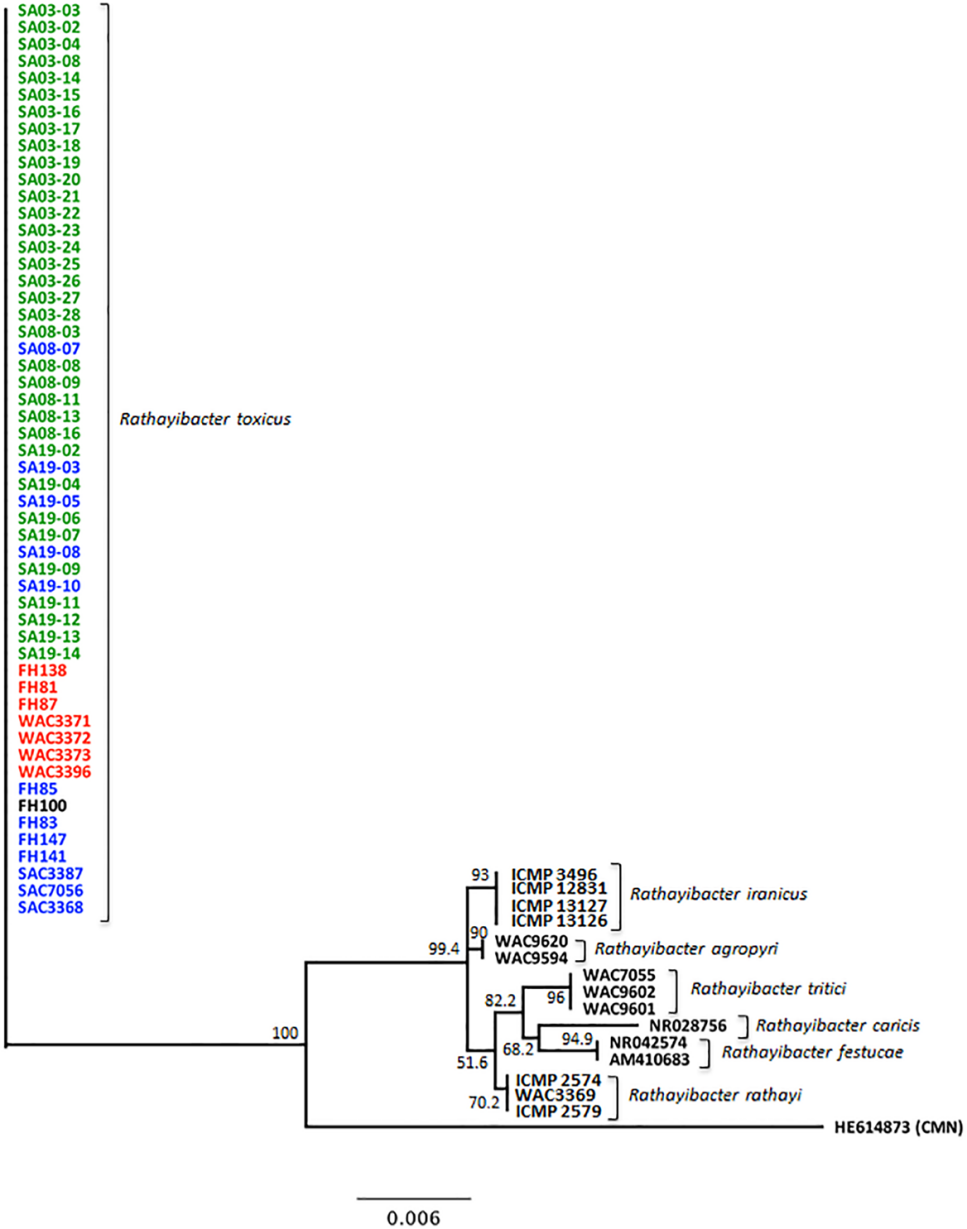
A phylogenetic tree was generated using consensus partial 16S ribosomal RNA gene sequence (about 1015 bp) of *Rathayibacter toxicus*, *R*. *tritici*, *R*. *agropyri*, *R*. *rathayi*, *R*. *iranicus*, *R*. *caricis* and *R*. *festucae*. ***Clavibacter michiganensis* subsp. *nebraskensis* was included as an outgroup.** The tree was constructed using neighbor-joining method and the Tamura-Nei genetic distance model. Detail of isolates and accession numbers of submitted sequences are given in Table 1 and S1 Table, respectively. A consensus tree was generated through bootstrap analysis using Geneious Tree Builder program with 1000 cycles; the obtained values labeled at the forks indicate the confidence limits for the grouping. The scale bar at the bottom indicates the substitution rate.

A 1110 bp fragment of the 16S ribosomal RNA gene was amplified from DNA of each isolate using primer set R16sF1 and R16sR1. A reliable, manually edited 1015 bp consensus sequence for each *R*. *toxicus* isolate was achieved after aligning the sense and anti-sense strands of partial sequence of the 16S ribosomal RNA gene. Consensus sequences of the 16S ribosomal RNA genes from isolates of *R*. *tritici*, *R*. *iranicus*, *R*. *rathayi* and *R*. *agropyri* were also obtained following the same procedure. All generated sequences (*R*. *toxicus*, *R*. *tritici*, *R*. *iranicus*, *R*. *rathayi*, *R*. *agropyri*) and sequences retrieved from the NCBI GenBank nucleotide database (*R*. *caricis*, *R*. *festucae* and *C*. *michiganensis* subsp. *nebraskensis*) were aligned and two independent trees using NJ (Fig 2) and UPGMA methods were generated (S1 Fig; Table 1 and S1 Table). There were no differences in the 16S rDNA nucleotide sequences among all *R*. *toxicus* isolates including those collected during 2013–2014 and those from archive collections. The *R*. *toxicus* 16S rDNA sequences showed 97.05, 97.34, 96.75, 96.95, 97.34 and 97.34% similarity with *R*. *tritici*, *R*. *agropyri*, *R*. *caricis*, *R*. *festucae*, *R*. *iranicus* and *R*. *rathayi*, respectively. *C*. *michiganensis* subsp. *nebraskensis*, an outgroup in this analysis, showed 94.42% similarity with *R*. *toxicus*. All generated 16S rDNA sequences were deposited in the NCBI GenBank nucleotide database (S1 Table).

### ISSR Analysis

**Fig 3:**
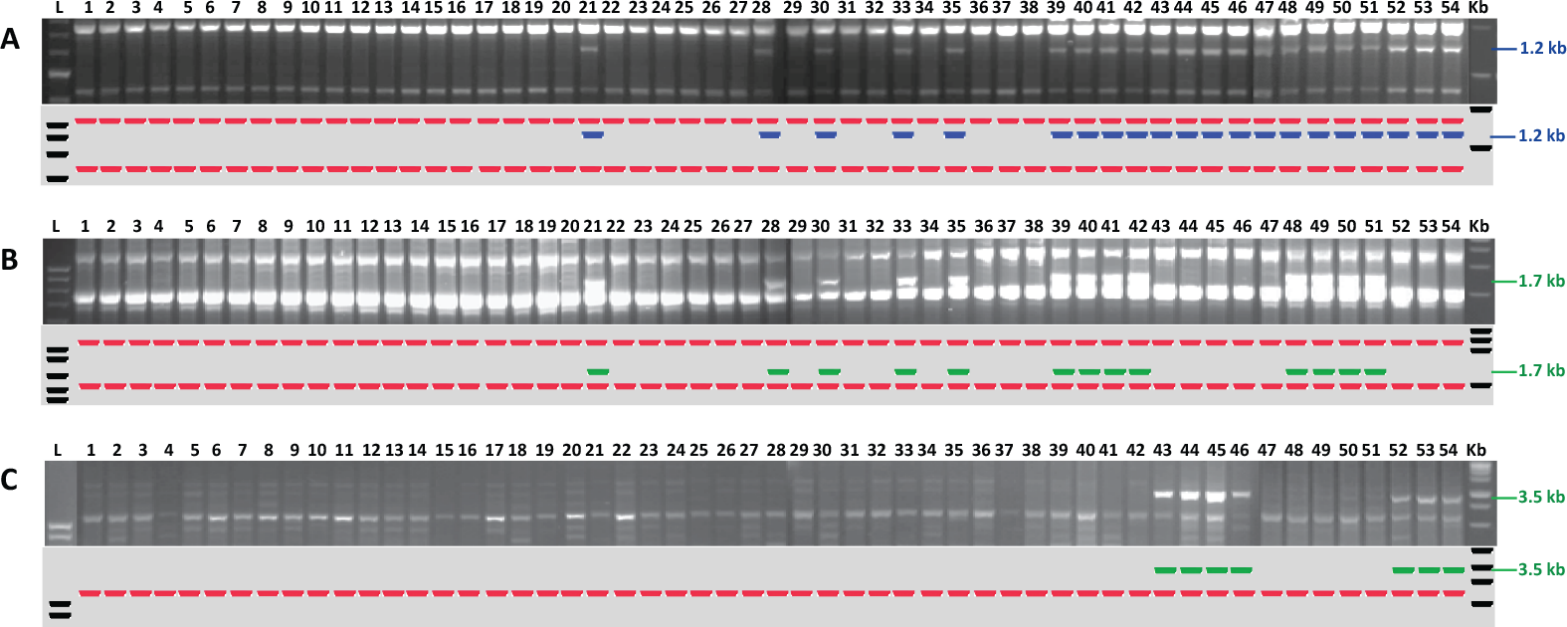
The ISSR profiles of 54 isolates of *Rathayibacter toxicus*. (A) Primer P15 produced an unique locus of 1.2 kb to differentiate population RT-I (locus absent) from RT-II and RT-III populations (locus present); (B) primer P16 produced an unique locus of 1.7 kb only with population RT-II; (C) primer UBC 810 produced an unique locus of 3.5 kb only with population RT-III of *R*. *toxicus*. The numbers above the gel images correspond to the individual isolates listed in Table 1.

**Fig 4:**
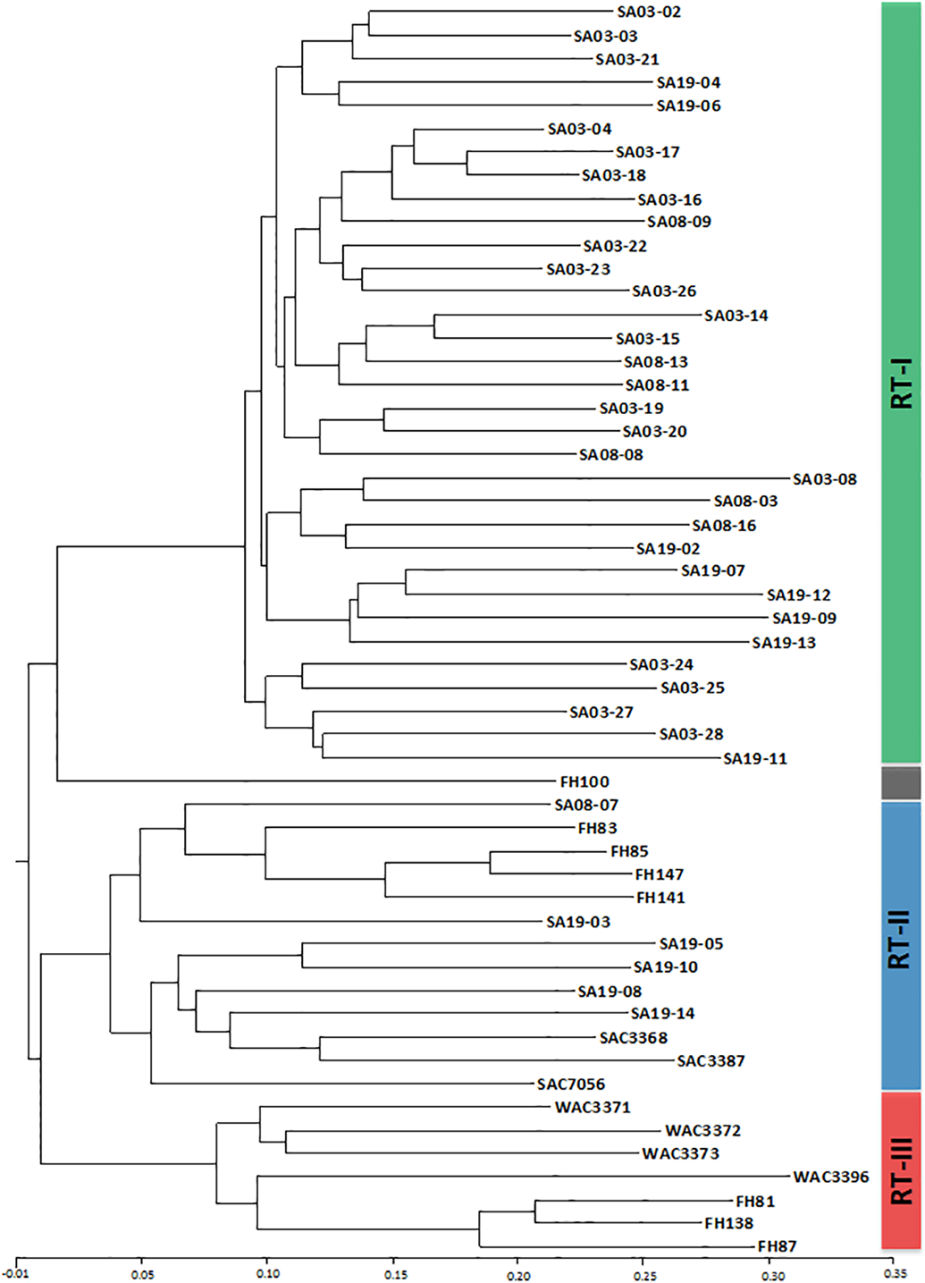
An ISSR phylogenetic tree of 54 isolates of *Rathayibacter toxicus* achieved using the neighbor-joining method. Isolates were grouped into three clusters named as population RT-I, RT-II and RT-III. The scale bar at the bottom indicates the dissimilarity among the isolates. Details for all isolates are listed in Table 1. This consensus tree was generated using bootstrap resampling method in Resample module of NTSYSpc with 1000 replicates.

A total of 10 ISSR primers (Table 3) amplified 94 loci including 65 polymorphic loci that accounted for 69% of the polymorphisms across the 54 isolates of *R*. *toxicus* (Table 1 and Table 3). The reproducibility of the ISSR results was verified by repeating the assays with selected *R*. *toxicus* isolates. The number of loci amplified from each ISSR primer ranged from 3 (UBC 881) to 17 (UBC 840) and the percentage polymorphism varied from 20 (P16) to 100 (UBC 810) (Table 3). Primers UBC 807 and UBC 991 each amplified one unique locus in isolates SAC3387 and SA03-08, respectively. Primer UBC 810 amplified one unique locus in isolate SA03-20 and one unique locus in isolate SA19-03 (Table 3). Since each unique locus was associated with single strain, it may be an artifact or signify a unique strain group. The presence of population specific unique loci enabled differentiation of populations RT-I, RT-II and RT-III (Fig 3). Based on ISSR analysis, the 54 *R*. *toxicus* isolates clustered into 3 major groups, denoted as populations RT-I, RT-II and RT-III (Fig 4). Population RT-I contained 33 isolates, population RT-II contained 14 isolates and population RT-III contained 7 isolates. All population RT-I isolates were collected in South Australia during the 2013–14 field survey. All population RT-III isolates were obtained from archive culture collections and were originally collected from Western Australia over 40 years. Six RT-II isolates were collected from South Australia during 2013–2014 while 8 RT-II isolates were obtained from archive culture collections collected from South Australia during 1973 to 2014.

Genetic similarity (Jaccard’s similarity coefficient) between isolates ranged from 64% (across populations) to 99% (within field collection site) based on ISSR analysis. None of the isolates showed 100% similarity with any other isolate. Jaccard’s similarity coefficient was 0.64 for isolates SA19-09 (Population RT-I) and WAC3396 (Population RT-III), 0.65 for isolates SA19-11 (Population RT-I) and WAC3396 (Population RT-III), 0.99 for isolates SA03-04 and SA03-18 (both Population RT-I), 0.99 for isolates SA03-17 and SA03-18 (both Population RT-I), and 0.99 for isolates FH85 and FH147 (both Population RT-II). PCOORDA of the ISSR data separated the *R*. *toxicus* isolates into the same populations (RT-I, RT-II, and RT-III) as the cluster analysis (S2 Fig). Isolate FH100, isolated from *P*. *monspeliensis* in 1991, showed some unique characteristics from the three populations but grouped with RT-II in the ISSR dendrogram generated using UPGMA method (S3 Fig). However, based on the PCOORDA and NJ, FH100 appeared distinct from RT-I, RT-II, and RT-III in the 2-D plot (S2 Fig) and NJ tree (Fig 4), respectively; the first three most informative PC components elucidated 51.58% of the total variation.

Analysis of molecular variance (AMOVA) among *R*. *toxicus* populations based on ISSR data indicated significant (P<0.001) genetic differentiation (*Φpt* value = 0.53); molecular variance among populations was 53% and within populations was 47%. Pairwise analyses between populations were RT-I vs RT-II (*Φpt* value = 0.563), RT-I vs RT-III (*Φpt* value = 0.695), and RT-II vs RT-III (*Φpt* value = 0.472). Percentages of polymorphic loci were 38% (RT-I), 47% (RT-II), and 27% (RT-III), with a mean value of 37% (SE 5.9%) (Table 4). There were 4, 5, and 4 unique loci (loci unique to a single population) identified in populations RT-I, RT-II and RT-III, respectively (Table 4). Pairwise population comparisons of Nei’s genetic distance was 0.151, 0.221 and 0.136 for population RT-I vs RT-II, RT-I vs RT-III, and RT-II vs RT-III, respectively. This indicates that highest genetic variation was between the RT-I and RT-III populations.

**Table 4:** ISSR loci data for the three *Rathayibacter toxicus* populations: RT-I, RT-II and RT-III.

Characteristics	Population		
	RT-I	RT-II	RT-III
Number of loci	82	86	72
% polymorphic loci	38%	47%	27%
Number of loci with a frequency > = 5%	76	86	72
No. of loci unique to a single population	4	5	4

### MLST Analysis

**Fig 5:**
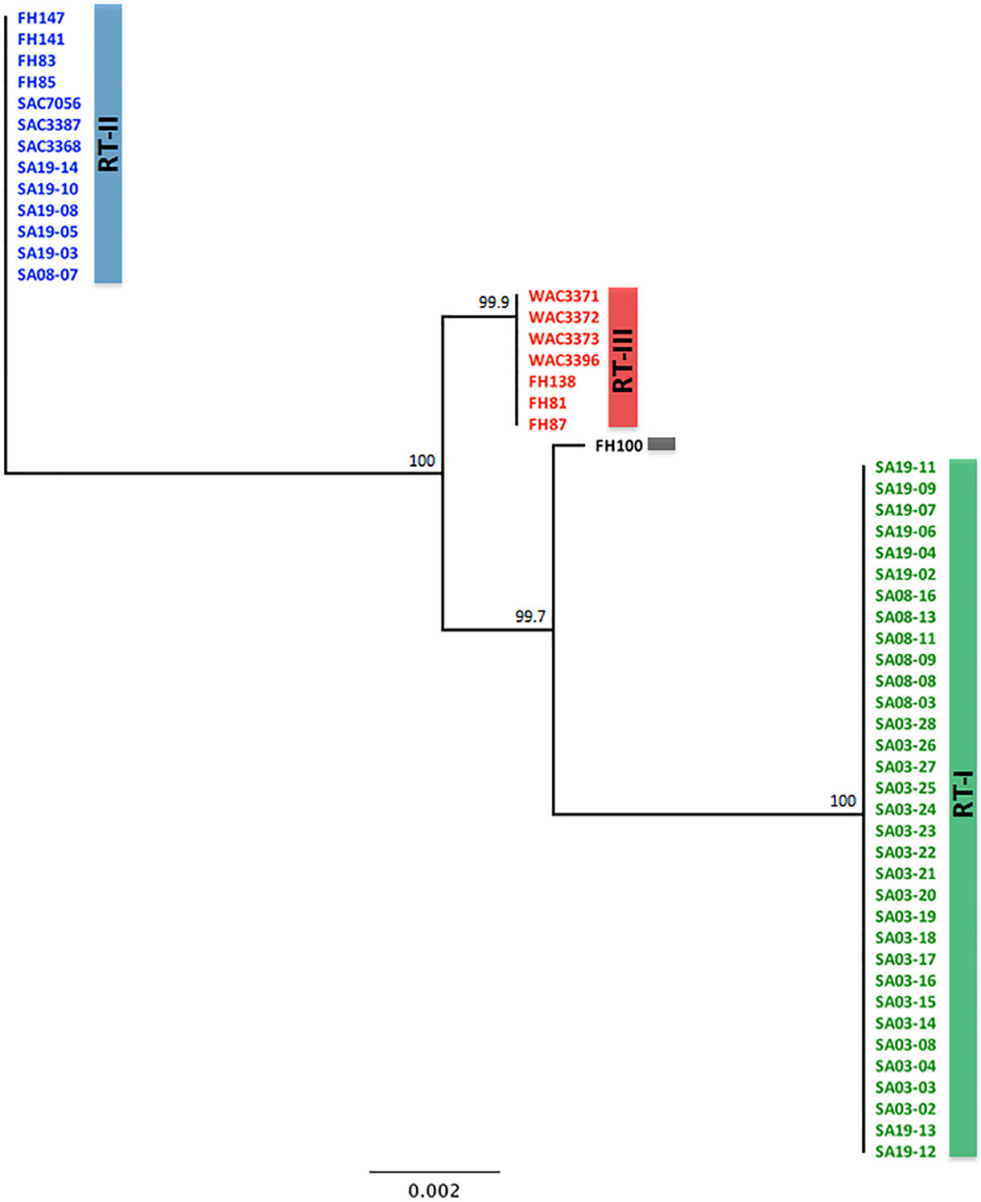
A phylogenetic tree of 54 isolates of *Rathayibacter toxicus* was generated using concatenated consensus partial gene sequences six genes. A total of 5,182 nucleotides from vancomycin resistant protein *vanA*, CRISPR-associated protein *cse4*, *secA* ATPase, chromosome partition protein SMC, tRNA dihydrouridine synthase, and cysteine desulfurase genes, were analyzed to generate this tree. Three distinct groups RT-I, RT-II and RT-II were formed. The tree was constructed using neighbor-joining and Tamura-Nei genetic distance model. A consensus tree was generated through bootstrap analysis using Geneious Tree Builder program with 1000 cycles; the obtained values labeled at the forks indicate the confidence limits for the grouping. The scale bar at the bottom indicates the substitution rate. Detail for all isolates and gene accession numbers submitted to NCBI GenBank are given in Table 1 and S1 Table.

Several conserved genes commonly used for MLST of bacterial populations: *rpoB* (sequence length 871 bp), *rpoD* (sequence length 867 bp), *dnaK* (sequence length 951 bp) and *gapA* (sequence length 894 bp) were screened for population differentiation. No differences in nucleotide sequences were detected for any of these conserved genes. For MLST analysis of these *R*. *toxicus* populations, genes were selected based on their discriminative power, cellular functions (acquired immunity, protein secretion, antibiotic resistance, chromosome condensation and partitioning, biosynthetic pathways and enzyme involve in dihydrouridine modification of tRNA), as well as spatial coverage of the entire genome (S4 Fig). Partial sequences of the following six genes were analyzed for all isolates: vancomycin resistant protein *vanA*, CRISPR-associated protein *cse4*, *secA* ATPase, chromosome partition protein SMC, tRNA dihydrouridine synthase and cysteine desulfurase (S4 Fig). The analyzed sequences comprised a total of 5,182 bp and accounted for 0.2% of the total 2.369 MB *R*. *toxicus* genome [40]. MLST analysis using these six genes resulted in the *R*. *toxicus* isolates NJ clustering into three populations RT-I, RT-II and RT-III (Fig 5). The percentage nucleotide difference for all six genes ranged from 0 (*secA* ATPase; between population RT-II and RT-III) to 2.5% (chromosome partition protein SMC; between population RT-I and RT-II; Table 5). A partial coding sequence of gene *secA*, which encodes an ATPase, contained one SNP that differentiated population RT-I from populations RT-II and RT-III, and FH100. Out of 5,182 nucleotides, the maximum nucleotide difference was 67 nucleotides between populations RT-I and RT-II, 40 nucleotides between RT-II and RT-III, and 39 nucleotides between RT-I and RT-III. For each MLST gene analyzed, there were no nucleotide differences among strains within a population. A dendrogram using UPGMA method was also generated; showed similar clustering (S5 Fig).

**Table 5:** Nucleotide differences in the partial gene sequences used for multi-locus sequence typing (MLST) analyses of *Rathayibacter toxicus* populations RT-I, RT-II, and RT-III.

Target Gene	Sequence length (bp)	Nucleotide difference					
		RT-I vs RT-II populations		RT-I vs RT-III populations		RT-II vs RT-III populations	
		Number	%	Number	%	Number	%
Chromosome partition protein SMC	912	23	2.5	11	1.2	18	2
tRNA dihydrouridine synthase	998	18	1.8	8	0.8	10	1
Cystein desulfurase	766	16	2.1	12	1.6	8	1
CRISPR-associated protein, *cse4* family	941	8	0.9	5	0.5	3	0.4
Vancomycin resistant protein *vanA*	831	1	0.1	2	0.2	1	0.1
*secA* ATPase	734	1	0.1	1	0.1	0	0
**Concatenated**	**5182**	**67**	**1.29**	**39**	**0.75**	**40**	**0.77**

**Fig 6:**
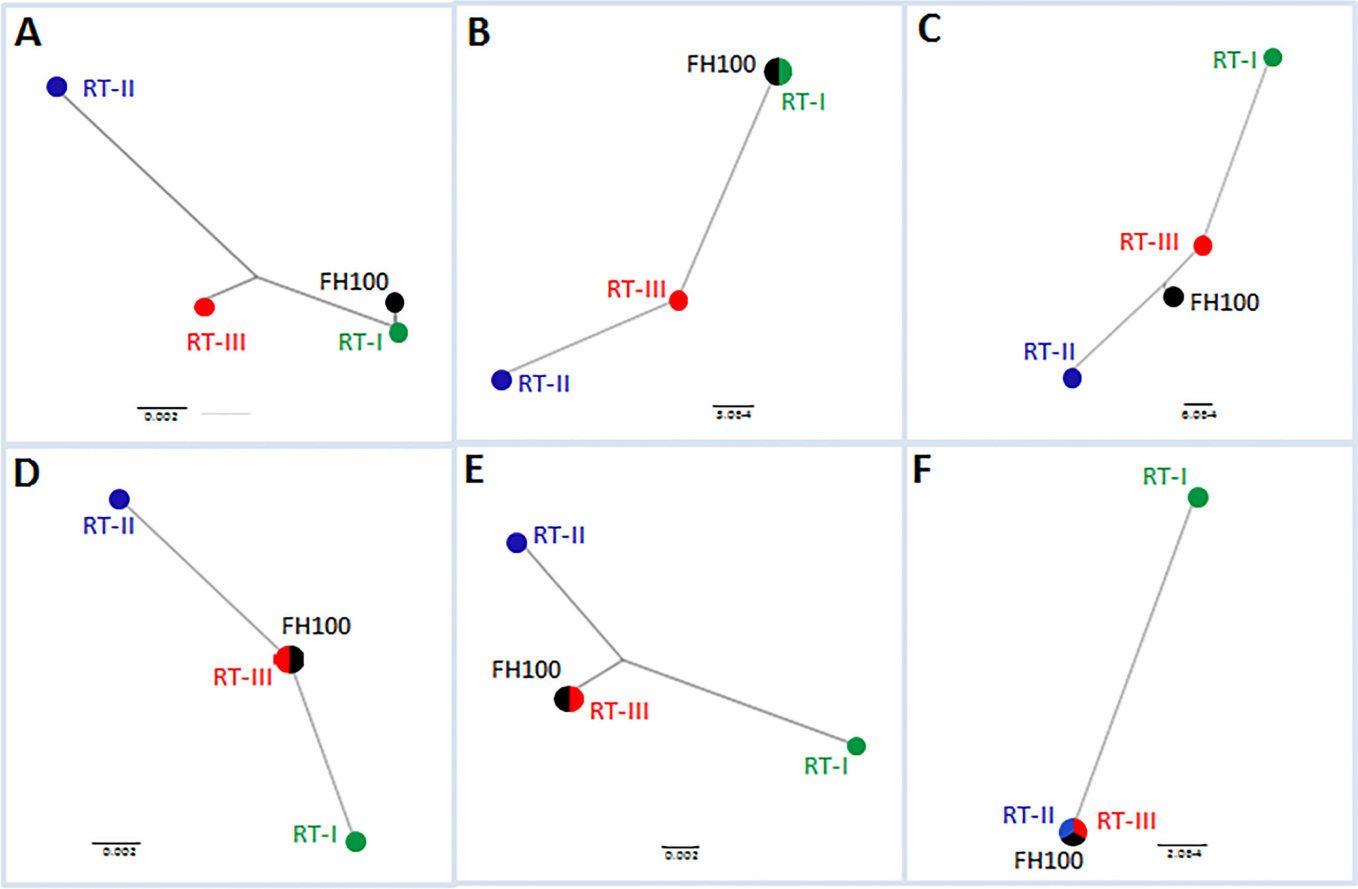
NJ phylogenetic trees were generated using consensus partial sequences of the individual genes used in the MLST analysis. (A) chromosome partition protein SMC; (B) vancomycin resistant protein *vanA*; (C) CRISPR-associated protein *cse4*; (D) tRNA dihydrouridine synthase; (E) cysteine desulfurase; and (F) *secA* ATPase. RT-I, RT-II and RT-III are the three populations of *R*. *toxicus* identified by MLST and ISSR. FH-100 is a single isolate that did not group consistently with all genes.

Individual gene analyses for all isolates resulted in similar clustering patterns except *secA* ATPase (Fig 6). However, isolate FH100 remained an anomaly. It clustered with or close to population RT-I when alignments of chromosome partition protein SMC (Fig 6A) and vancomycin resistant protein were used (Fig 6B); FH100 clustered with or close to population RT-III when using the CRISPR-associated protein gene *cse4* (Fig 6C), tRNA dihydrouridine synthase (Fig 6D) and cysteine desulfurase (Fig 6E) genes. The *secA* ATPase gene formed only two groups (group 1 with population RT-I and group 2 that combined populations RT-II and RT-III); FH100 clustered with 2^nd^ group for the *secA* ATPase gene (Fig 6F). When the sequences of all six MLST genes were concatenated and analyzed, FH100 clustered close to population RT-III but remain distinct (Fig 6). Trees of the individual genes were also generated using UPGMA method; they showed similar clustering patterns as the NJ method (S6 Fig).

## Discussion

A new population of the U.S. select agent *R*. *toxicus* (RT-I) was detected in 2013–2014 from the Yorke Peninsula in South Australia. RT-I was the predominant population on the Yorke Peninsula during our sampling surveys. At two of the three *R*. *toxicus*-positive sampling sites on the Yorke Peninsula, population RT-II was also detected along with RT-I. Sampling over the 40 years prior to 2013–2014 failed to detect the RT-I population. Possible explanations include: 1) the sampling strategy did not fully cover the geographic distribution of *R*. *toxicus* on the Yorke Peninsula, 2) RT-I was present but the sampling protocol was not sensitive to low frequency genotypes, or 3) the RT-I population recently emerged on the Yorke Peninsula. Genetic variation among isolates within the RT-I population as indicated in the ISSR analysis was much less than the genetic variation among isolates within the RT-II and the RT-III populations, perhaps suggesting a more recent emergence of RT-I. It is possible that failure to detect the RT-I population during early surveys was due to sampling error; sampling method or very low RT-I prevalence. At low population densities, the spatial distribution of *R*. *toxicus* is patchy within fields [41]. At two South Australia sample sites (SA08 and SA19) in this study, RT-II was present at low incidence compared to RT-I. Yet, both the RT-I and RT-II populations were isolated from both sites (Table 1; Fig 4) indicating that the sampling protocol was sensitive to detecting genotypes at low population densities with patchy distributions. This is consistent with prior research [16, 17]. Consequently, if RT-I was present in South Australia during the surveys of the past 40 years, the probability of detection was reasonable supporting a more recent emergence of RT-I. It is possible that this genotype existed a long time ago but was only recently disseminated across the Yorke Peninsula either through natural weather events, via the movement of infected seed or infested hay, or on farm equipment. That RT-I has become the dominant population might suggest some competitive advantage at least on the Yorke Peninsula. Comparative genomic analyses currently underway may provide more insight into the origin and evolution of RT-I. The RT-I genome [40] does contain the cluster of toxin producing genes associated with tunicamycin synthesis (J. P. Stack; unpublished information).

Only three out of 29 sites visited during the sample collection in 2013–2014 were positive for *R*. *toxicus*. The low number of positive sites may have been due to aggressive ryegrass management practices or environmental conditions in the previous year. The spatial distribution of *R*. *toxicus* as well as the irregular occurrence of outbreaks of *R*. *toxicus*-induced toxicities, have been reported to be patchy [41]. The identity of each isolate was confirmed using partial sequence (1015 bp) of the 16S ribosomal gene, a gene commonly used for bacterial identification and to identify phylogenetic relationships [42, 43]. All 54 isolates from the RT-I, RT-II and RT-III populations had 100% homology in the 16S ribosomal gene region and thus were confirmed as *R*. *toxicus* (Fig 2).

ISSR and MLST are commonly used methods for phylogenetic studies [21, 24, 26, 28]. Several commonly used MLST gene targets including *rpoB*, *rpoD*, *dnaK* and *gapA* did not discriminate among the fifty-four *R*. *toxicus* isolates. Had only those gene targets been used, the conclusion would have been that no variation existed among the Australian *R*. *toxicus* populations as a function of geography or time. Criteria for MLST gene selection and a core MLST gene set was proposed for members of the subclass *Actinobacteridae* and included, *ychF* (putative DNA-binding GTPase), *rpoB* (β subunit of bacterial RNA polymerase), and *secY* (subunit of Type II secretory pathway ATPase) gene targets [44]. However, in this study- *rpoB* and *rpoD* partial gene sequences were not informative at the population level of discrimination. All the genes including *secA* partial gene sequence with a single SNP were informative for *R*. *toxicus* at the population level; a single *secA* SNP was common to all RT-I isolates from three sample locations across a sampling area of 55 kilometers and different from all isolates of populations RT-II, RT-III and FH100 (Fig 6F). The six gene targets reported here were strategically selected to represent an array of cellular functions: acquired immunity, protein secretion, antibiotic resistance, chromosome condensation and partitioning, biosynthetic pathways and enzyme involve in dihydrouridine modification of tRNA. Whereas no variation was observed with commonly used conserved genes, the six-gene MLST reported here resolved three populations of *R*. *toxicus* as a function of geography and time. Similar experience was reported with *Xylella fastidiosa* where standard house-keeping genes failed to identify genetic variation while a multi-locus sequence analysis based upon environmentally-mediated genes (MLSA-E; environmental sensitive genes) resolved variation and revealed relationships among closely related bacterial strains [45].

The ISSR markers were able to discriminate isolates based on geographical regions. In our study, ISSR primers produced signature profiles that grouped isolates based on their geographic origin (Fig 4 and S2 Fig) and could be used in trace back studies for applications in plant biosecurity. Ten polymorphic ISSR primers were selected for the analysis of *R*. *toxicus* isolates and amplified 94 loci including 69% polymorphic loci (Table 3). The ISSR analysis grouped the isolates into three clusters with the exception of isolate FH100 (Fig 4 and S2 Fig). However, FH100 was isolated from a different host, *P*. *monspeliensis*, and different geographic area of South Australia. Similar results were obtained by Agarkova et al. [16] where 22 strains of *R*. *toxicus* were grouped into two clusters and FH100 was separate from these clusters. In our analyses, ISSR showed better resolution among the *R*. *toxicus* isolates within populations compared to MLST (Figs 4 and 5 and S2 Fig). Baysal et al. [21] used ISSR method to effectively track the strains of *C*. *michiganensis* subsp. *michiganesis* isolates in Southern Turkey.

Both ISSR and MLST supported the existence of three distinct populations RT-I, RT-II and RT-III of *R*. *toxicus*. Although the MLST genes were distributed across the entire *R*. *toxicus* genome, the better resolution afforded by ISSR compared to MLST may have been the result of a more complete genome coverage of *R*. *toxicus*. Therefore, a more analytical approach for identifying informative genome regions for MLST may be necessary to finely resolve population structure in some pathogenic bacteria [46]. This result also supports the value of pan-genomic analysis for the identification of gene targets of value to ecological investigations that require the identification of pathogenic bacteria at sub-specific levels of discrimination (e.g., race, pathovar, biovar) [47].

Consistent with the results obtained in this study, in all previous *R*. *toxicus* population genetic studies, isolates from Western Australia grouped independently from isolates from South Australia [16, 17]. In those studies, genetic variation within the Western Australia population and within the South Australian population was identified using several analytical approaches including isozyme analysis, amplified fragment length polymorphisms, and pulsed-field gel electrophoresis [16, 17]. Within-population genetic variation among the Western Australia isolates was not correlated to isolation location, host or date of isolation suggesting that these populations are derived from one to a few clonal lineages [17]. The *R*. *toxicus* isolates used in those studies were all collected approximately 25–40 years ago, excluding one isolate from 2001 that showed no genetic variation from previously characterized Western Australia isolates [16]. In this study, fifty-four isolates of *R*. *toxicus* collected over a period of 40 years from South Australia and Western Australia were resolved into three distinct genetic groups by two independent analyses, a neutral-locus ISSR method and a coding sequence-based MLST. Sample integrity was preserved from field to lab and all *R*. *toxicus* isolates were cultured from individual bacterial galls collected from infected, mature annual ryegrass heads and processed to preclude cross contamination. In this and previous studies, the same basic result was obtained; various cluster analyses grouped the Western Australia isolates distinct from the South Australia isolates. In all studies, genetic variation within populations was identified. Several isolates were common to this and a previous study [16]; isolates that grouped together in this study by ISSR and sequence-based MLST also grouped together in the previous study by AFLP and PFGE. Of note in this study, a previously unreported population (RT-I) was detected in South Australia and was the dominant genotype detected on the Yorke Peninsula in 2013–2014. Isolates in group RT-I were genetically distinct from all *R*. *toxicus* isolates previously reported.

One isolate, FH100, was reported by Agarkova et al. [16] as an outlier to the other two groups (Western Australia and South Australia). DNA of FH100 was provided by the Vidaver laboratory and included in this study. Consistent with their findings, FH100 presented as a single isolate cluster in this study when the concatenated MLST sequence was analyzed. When individual-gene trees were generated (Fig 6), FH100 grouped with population RT-I, RT-II, or RT-III, depending upon the gene. This isolate was cultured from *P*. *monspeliensis* collected in southeast South Australia in 1991 [16]; a different host species and different location than the other isolates. Whether this is evidence of another distinct *R*. *toxicus* population can only be confirmed by analyzing additional isolates from this host and location; they were not available for this study. Although in all studies to date, isolates did group as a function of geographic origin at a macro spatial scale (Western Australia and South Australia), no correlation has been reported between genetic variation and geographic origin at lesser spatial scales [17].

In this communication we report the emergence and establishment of a new population of *R*. *toxicus* on the Yorke Peninsula of South Australia. The global trade of ryegrass seed and hay makes possible the potential extension of the geographic range of *R*. *toxicus* through the dissemination of these commodities. Extension to new geographic areas may pose a threat to animal health and provide new evolutionary opportunities for the pathogen. Understanding the nature and magnitude of genetic variation in *R*. *toxicus* will provide insight into its life history, center of origin and evolutionary potential.

## Supporting Information

S1 Fig An UPGMA phylogenetic tree was generated using consensus partial 16S ribosomal RNA gene sequence of *Rathayibacter toxicus*, *R*. *tritici*, *R*. *agropyri*, *R*. *rathayi*, *R*. *iranicus*, *R*. *caricis* and *R*. *festucae*. ***Clavibacter michiganensis* subsp. *nebraskensis* was included as an outgroup.** The tree was constructed using UPGMA (unweighted pair-group method with arithmetic mean) method. Detail of isolates and accession numbers of submitted sequences are given in Table 1 and S1 Table, respectively. A consensus tree was generated through bootstrap analysis using Geneious Tree Builder program with 1000 cycles; the obtained values labeled at the forks indicate the confidence limits for the grouping. The scale bar at the bottom indicates the dissimilarity.
(TIF)

S2 Fig Two-dimensional vector plot of 54 isolates of *Rathayibacter toxicus* achieved using principal coordinate analysis (PCOORDA) of ISSR data. Three distinct groups RT-I, RT-II and RT-II were formed. Isolate FH-100 grouped independently from all the other isolates of *R*. *toxicus*. The numbers correspond to the individual isolates listed in Table 1.
(TIF)

S3 Fig An ISSR phylogenetic tree of 54 isolates of *Rathayibacter toxicus* achieved using UPGMA based on Jaccard’s coefficient. Isolates were grouped into three clusters named as population RT-I, RT-II and RT-III. The scale bar at the bottom indicates the similarity coefficient among the isolates. Details for all isolates are listed in Table 1. Consensus tree was generated using bootstrap resampling method in Resample module of NTSYSpc with 1000 replicates.
(TIF)

S4 Fig Locations of gene sequences across the complete genome of *Rathayibacter toxicus* (A; SA03-04). Six genes viz. vancomycin resistant protein *vanA* (C), CRISPR-associated protein *cse4* (D), *secA* ATPase (F), chromosome partition protein SMC (G), tRNA dihydrouridine synthase (H), and cystein desulfurase (I) were used in multi-locus sequence typing (MLST). Two copies of the 16S ribosomal RNA gene (B and E) are present in the *R*. *toxicus* genome. Partial sequence of the 16S ribosomal RNA gene was used to confirm the identity of the isolates to species. Color codes: red indicates the target gene; crimson (dark red) delimited by vertical lines indicates the segments of the target gene that were amplified and used to generate the trees; green represents the portion of the genome that was not used in our study.
(TIF)

S5 Fig An UPGMA phylogenetic tree of 54 isolates of *Rathayibacter toxicus* was generated using concatenated consensus partial gene sequences of six genes. A total of 5,182 nucleotides from vancomycin resistant protein *vanA*, CRISPR-associated protein *cse4*, *secA* ATPase, chromosome partition protein SMC, tRNA dihydrouridine synthase, and cysteine desulfurase genes, were analyzed to generate this tree. Three similar distinct groups RT-I, RT-II and RT-III were formed as using the NJ method. The tree was constructed using UPGMA (unweighted pair-group method with arithmetic mean) method. A consensus tree was generated through bootstrap analysis using Geneious Tree Builder program with 1000 cycles; the obtained values labeled at the forks indicate the confidence limits for the grouping. The scale bar at the bottom indicates the dissimilarity. Detail for all isolates and gene accession numbers submitted to NCBI GenBank are given in Table 1 and S1 Table.
(TIF)

S6 Fig UPGMA phylogenetic trees were generated using consensus partial sequences of the individual genes used in the MLST analysis. (A) chromosome partition protein SMC; (B) vancomycin resistant protein *vanA*; (C) CRISPR-associated protein *cse4*; (D) tRNA dihydrouridine synthase; (E) cysteine desulfurase; and (F) *secA* ATPase. RT-I, RT-II and RT-III are the three populations of *R*. *toxicus* identified by MLST and ISSR. FH100 is a single isolate that did not group consistently with all genes.
(TIF)

S1 Table Gene sequences of bacterial isolates used in this study were submitted to NCBI GenBank nucleotide database under the accession numbers mentioned in this Table. (DOCX)
