# Supplementary figures and images for: Emergence of a New Population of Rathayibacter toxicus: An Ecologically Complex, Geographically Isolated Bacterium
Source: PLoS One. 2016 May 24;11(5):e0156182. doi: 10.1371/journal.pone.0156182 (PMC4878776; doi:10.1371/journal.pone.0156182)

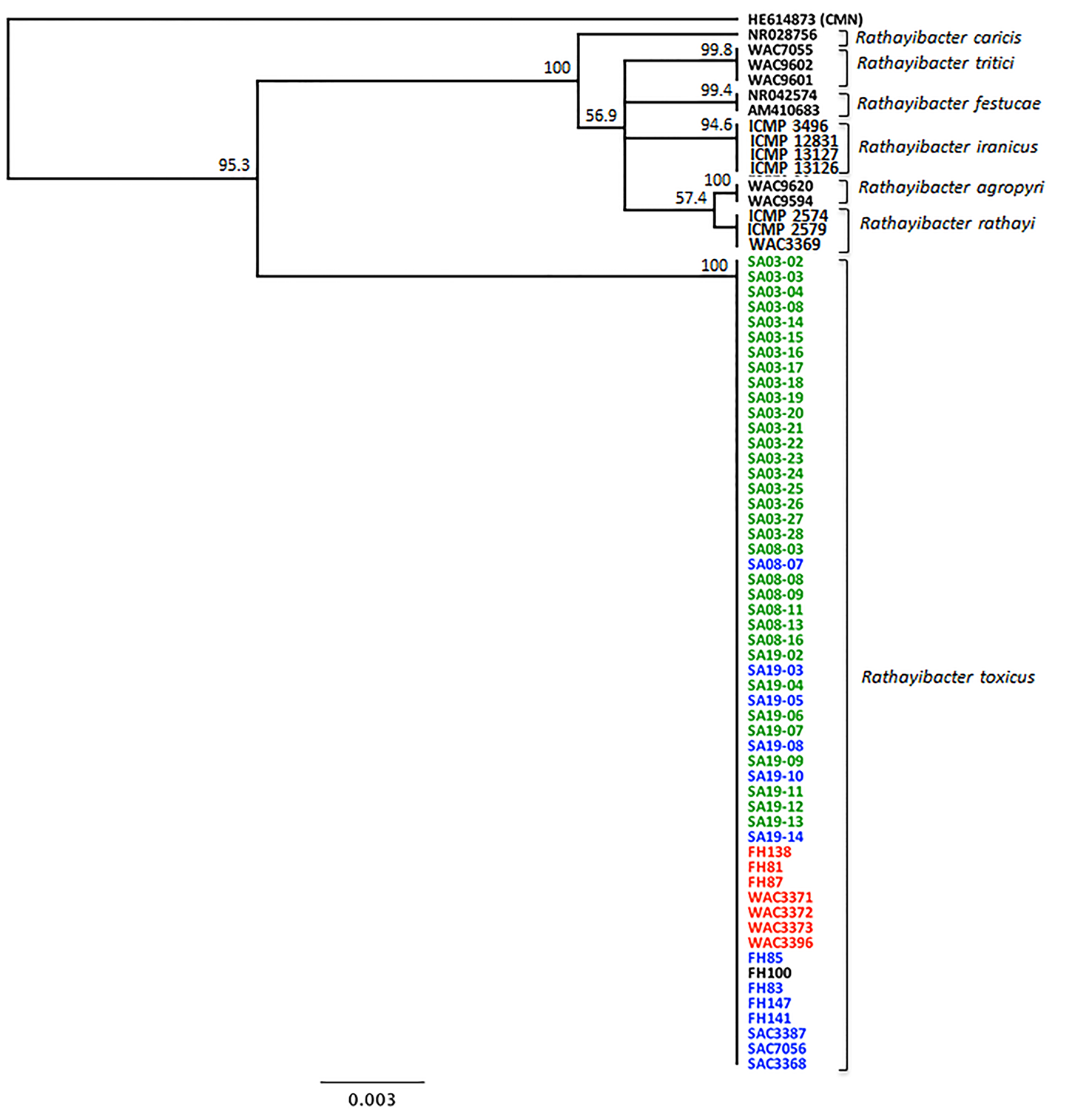

Supplement: S1 Fig — Clavibacter michiganensis subsp. nebraskensis was included as an outgroup. The tree was constructed using UPGMA (unweighted pair-group method with arithmetic mean) method. Detail of isolates and accession numbers of submitted sequences are given in Table 1 and S1 Table, respectively. A consensus tree was generated through bootstrap analysis using Geneious Tree Builder program with 1000 cycles; the obtained values labeled at the forks indicate the confidence limits for the grouping. The scale bar at the bottom indicates the dissimilarity. (TIF) [file pone.0156182.s001.tif]

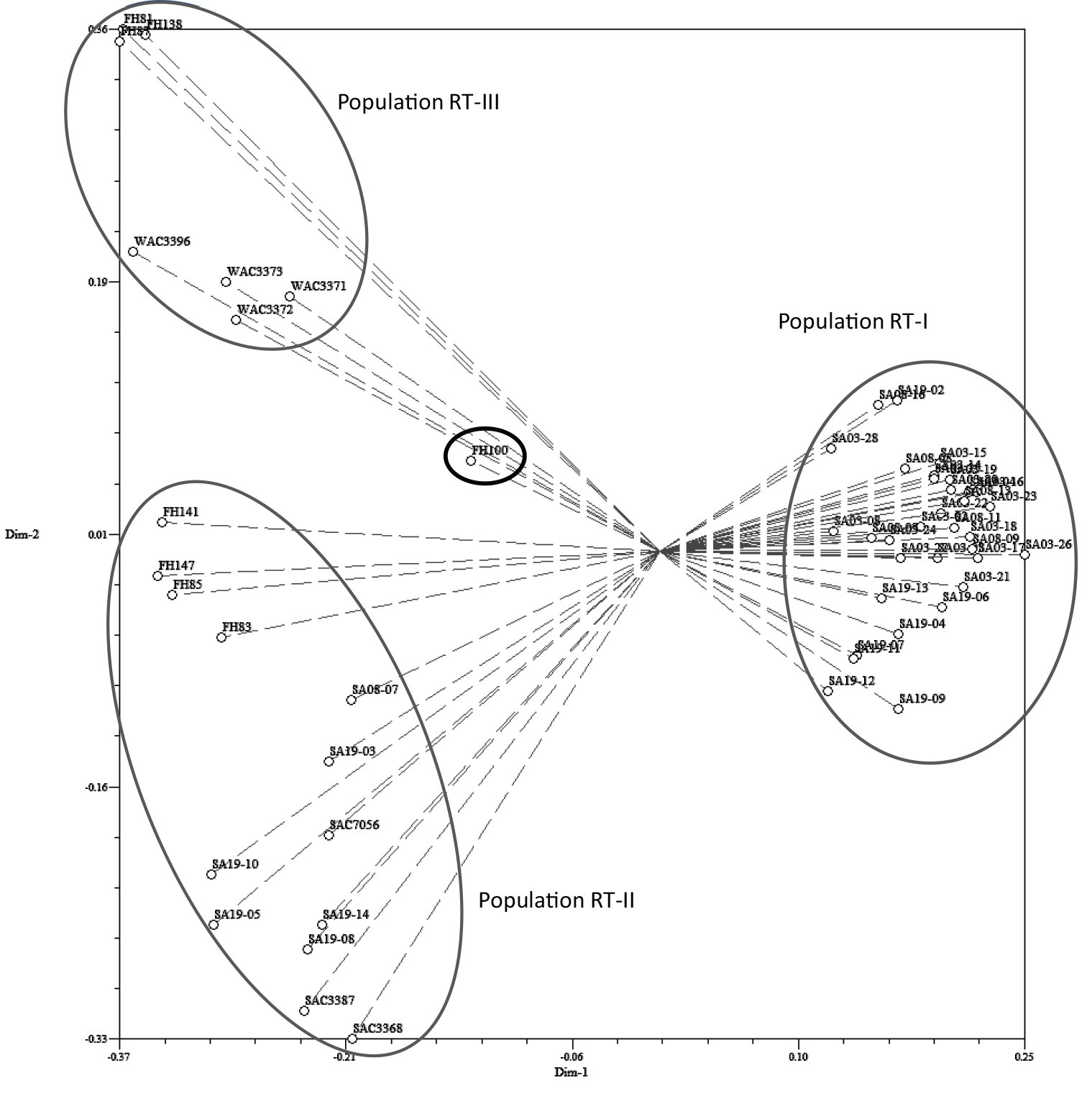

Supplement: S2 Fig — Three distinct groups RT-I, RT-II and RT-II were formed. Isolate FH-100 grouped independently from all the other isolates of R. toxicus. The numbers correspond to the individual isolates listed in Table 1. (TIF) [file pone.0156182.s002.tif]

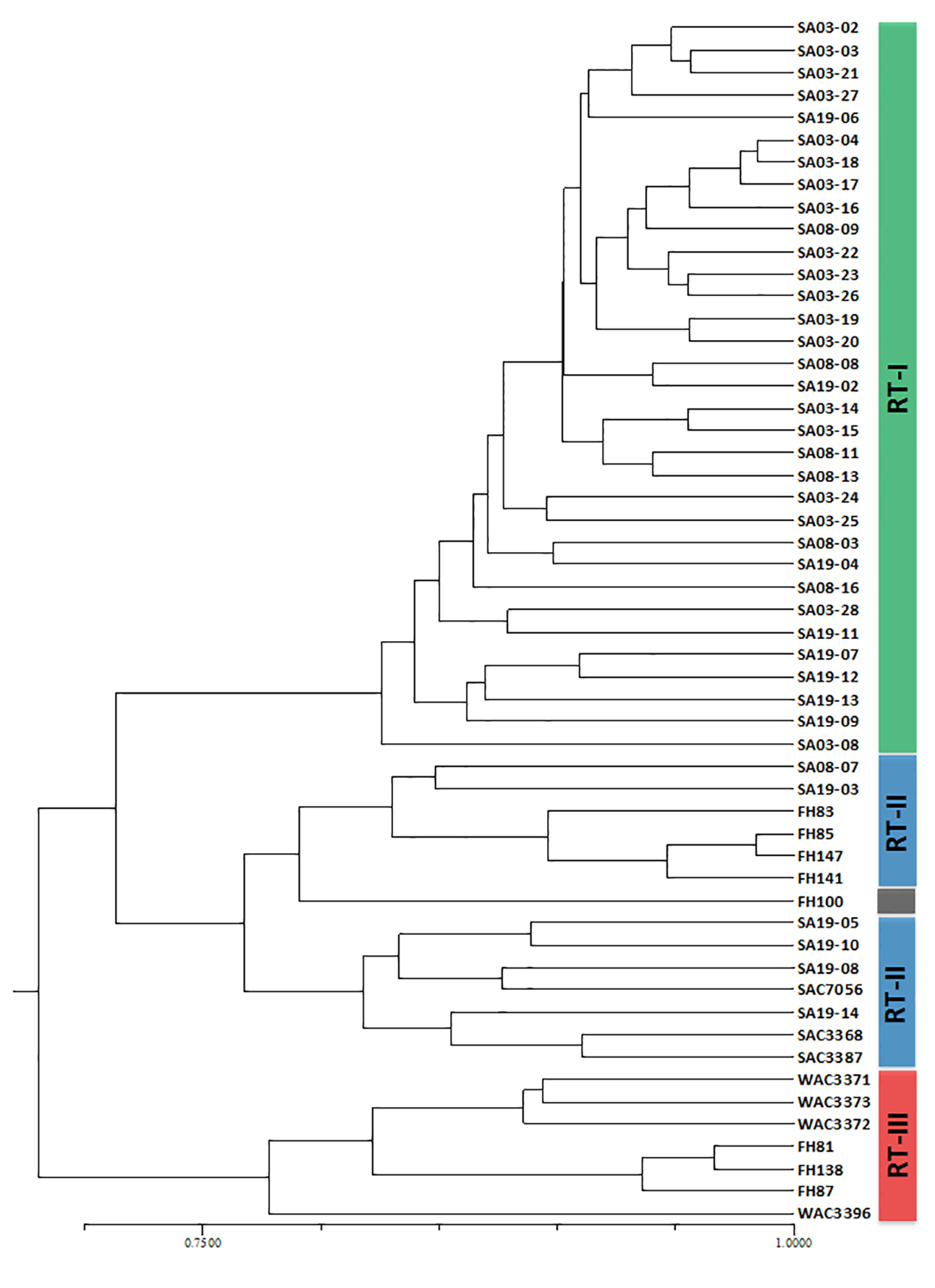

Supplement: S3 Fig — Isolates were grouped into three clusters named as population RT-I, RT-II and RT-III. The scale bar at the bottom indicates the similarity coefficient among the isolates. Details for all isolates are listed in Table 1. Consensus tree was generated using bootstrap resampling method in Resample module of NTSYSpc with 1000 replicates. (TIF) [file pone.0156182.s003.tif]

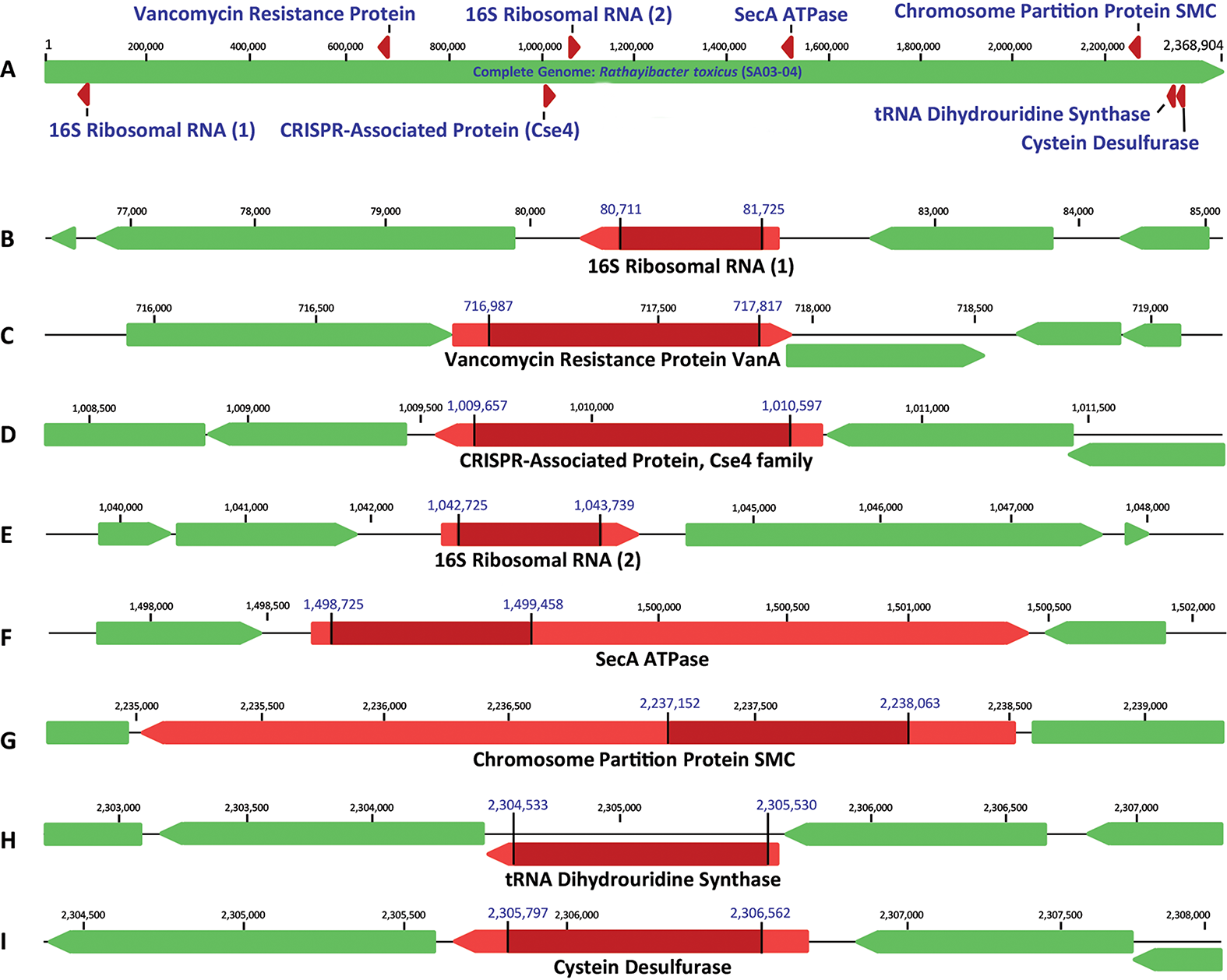

Supplement: S4 Fig — Six genes viz. vancomycin resistant protein vanA (C), CRISPR-associated protein cse4 (D), secA ATPase (F), chromosome partition protein SMC (G), tRNA dihydrouridine synthase (H), and cystein desulfurase (I) were used in multi-locus sequence typing (MLST). Two copies of the 16S ribosomal RNA gene (B and E) are present in the R. toxicus genome. Partial sequence of the 16S ribosomal RNA gene was used to confirm the identity of the isolates to species. Color codes: red indicates the target gene; crimson (dark red) delimited by vertical lines indicates the segments of the target gene that were amplified and used to generate the trees; green represents the portion of the genome that was not used in our study. (TIF) [file pone.0156182.s004.tif]

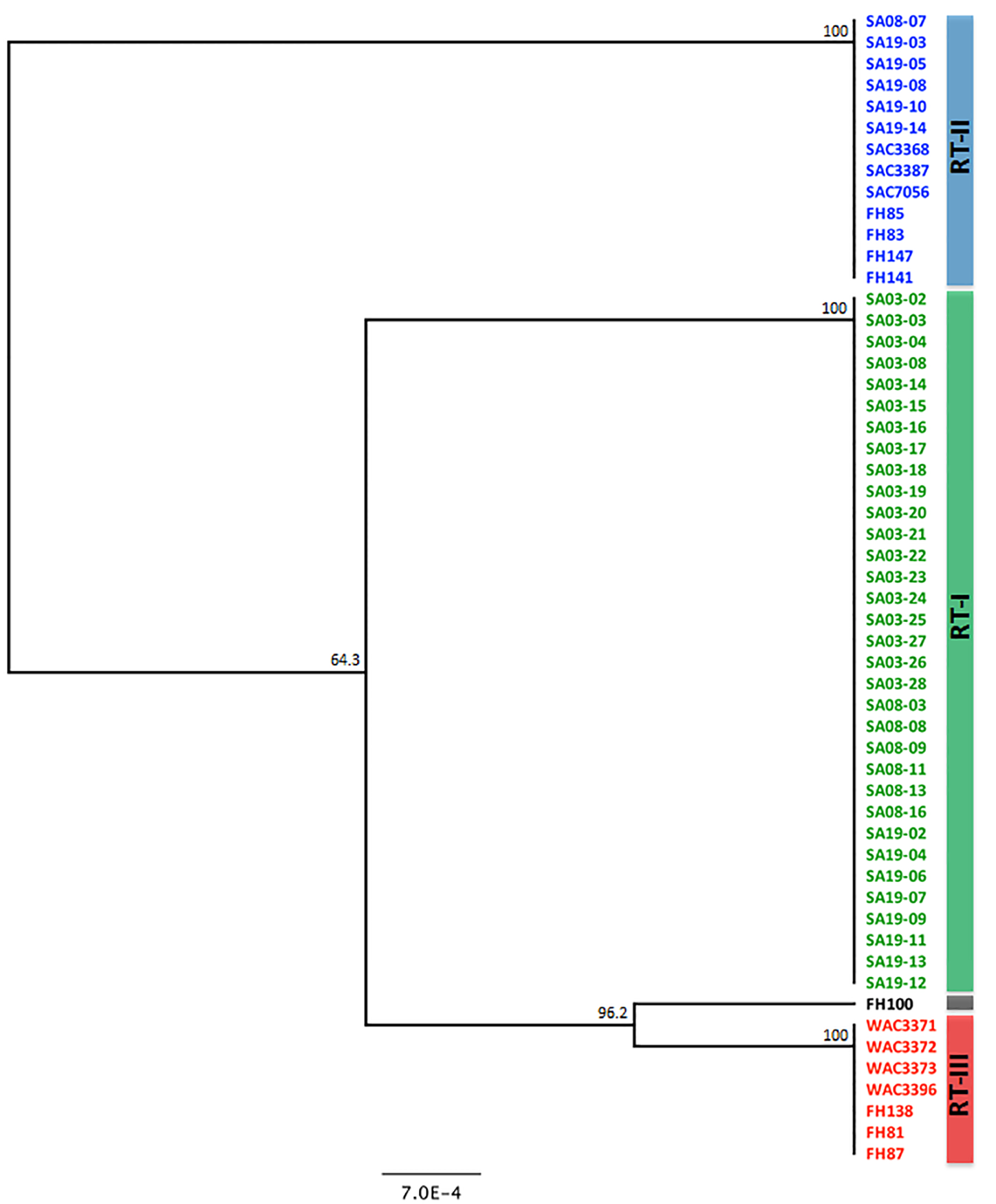

Supplement: S5 Fig — A total of 5,182 nucleotides from vancomycin resistant protein vanA, CRISPR-associated protein cse4, secA ATPase, chromosome partition protein SMC, tRNA dihydrouridine synthase, and cysteine desulfurase genes, were analyzed to generate this tree. Three similar distinct groups RT-I, RT-II and RT-III were formed as using the NJ method. The tree was constructed using UPGMA (unweighted pair-group method with arithmetic mean) method. A consensus tree was generated through bootstrap analysis using Geneious Tree Builder program with 1000 cycles; the obtained values labeled at the forks indicate the confidence limits for the grouping. The scale bar at the bottom indicates the dissimilarity. Detail for all isolates and gene accession numbers submitted to NCBI GenBank are given in Table 1 and S1 Table. (TIF) [file pone.0156182.s005.tif]

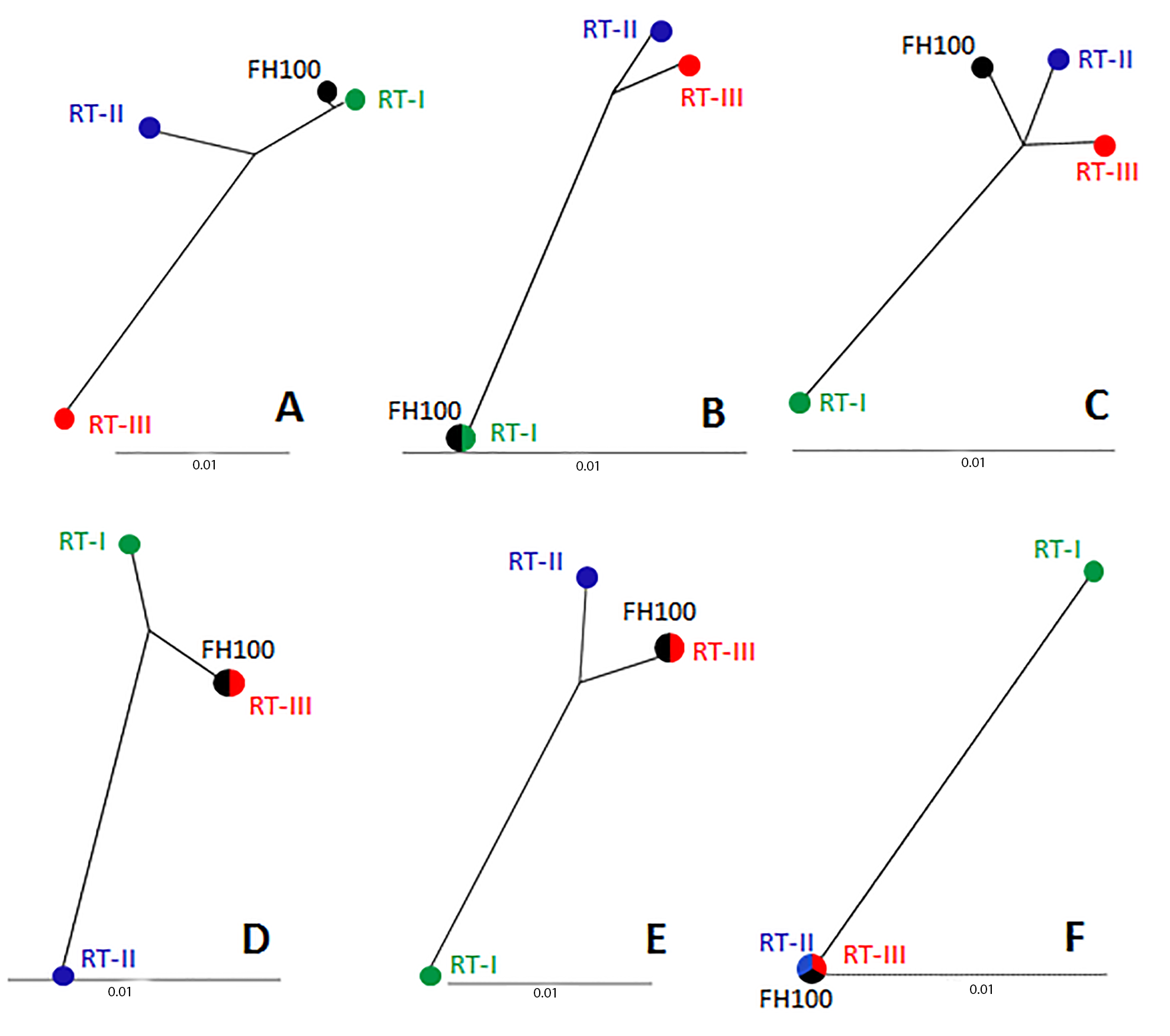

Supplement: S6 Fig — (A) chromosome partition protein SMC; (B) vancomycin resistant protein vanA; (C) CRISPR-associated protein cse4; (D) tRNA dihydrouridine synthase; (E) cysteine desulfurase; and (F) secA ATPase. RT-I, RT-II and RT-III are the three populations of R. toxicus identified by MLST and ISSR. FH100 is a single isolate that did not group consistently with all genes. (TIF) [file pone.0156182.s006.tif]
