# Supplementary material for: Emergence of a New Population of Rathayibacter toxicus: An Ecologically Complex, Geographically Isolated Bacterium
Source: PLoS One. 2016 May 24;11(5):e0156182. doi: 10.1371/journal.pone.0156182 (PMC4878776; doi:10.1371/journal.pone.0156182)
Supplement: S1 Table — (DOCX) [file pone.0156182.s007.docx]

**S1 Table. Gene sequences of bacterial isolates used in this study were submitted to NCBI GenBank nucleotide database under the accession numbers mentioned in this Table.**

| Genus/Species | Isolate Code | Target Genes | | | | | | |
| --- | --- | --- | --- | --- | --- | --- | --- | --- |
|  |  | 16S Ribosomal RNA | Vancomycin A-type Resistant Protein *vanA* | CRISPR-Associated Protein, *cse4* | *secA* ATPase | Chromosome Partition Protein SMC | tRNA Dihydrouridine Synthase | Cysteine Desulfurase |
| *Rathayibacter toxicus* | SA03-02 | KT760418.1 | KT875683 | KT875413 | KT875629.1 | KT875521.1 | KT875575.1 | KT875467.1 |
| *R. toxicus* | SA03-03 | KT760419.1 | KT875684.1 | KT875414.1 | KT875630.1 | KT875522.1 | KT875576.1 | KT875468.1 |
| *R. toxicus* | SA03-04 | KT760420.1 | KT875685.1 | KT875415.1 | KT875631.1 | KT875523.1 | KT875577.1 | KT875469.1 |
| *R. toxicus* | SA03-08 | KT760421.1 | KT875686.1 | KT875416.1 | KT875632.1 | KT875524.1 | KT875578.1 | KT875470.1 |
| *R. toxicus* | SA03-14 | KT760422.1 | KT875687.1 | KT875417.1 | KT875633.1 | KT875525.1 | KT875579.1 | KT875471.1 |
| *R. toxicus* | SA03-15 | KT760423.1 | KT875688.1 | KT875418.1 | KT875634.1 | KT875526.1 | KT875580.1 | KT875472.1 |
| *R. toxicus* | SA03-16 | KT760424.1 | KT875689.1 | KT875419.1 | KT875635.1 | KT875527.1 | KT875581.1 | KT875473.1 |
| *R. toxicus* | SA03-17 | KT760425.1 | KT875690.1 | KT875420.1 | KT875636.1 | KT875528.1 | KT875582.1 | KT875474.1 |
| *R. toxicus* | SA03-18 | KT760426.1 | KT875691.1 | KT875421.1 | KT875637.1 | KT875529.1 | KT875583.1 | KT875475.1 |
| *R. toxicus* | SA03-19 | KT760427.1 | KT875692.1 | KT875422.1 | KT875638.1 | KT875530.1 | KT875584.1 | KT875476.1 |
| *R. toxicus* | SA03-20 | KT760428.1 | KT875693.1 | KT875423.1 | KT875639.1 | KT875531.1 | KT875585.1 | KT875477.1 |
| *R. toxicus* | SA03-21 | KT760429.1 | KT875694.1 | KT875424.1 | KT875640.1 | KT875532.1 | KT875586.1 | KT875478.1 |
| *R. toxicus* | SA03-22 | KT760430.1 | KT875695.1 | KT875425.1 | KT875641.1 | KT875533.1 | KT875587.1 | KT875479.1 |
| *R. toxicus* | SA03-23 | KT760431.1 | KT875696.1 | KT875426.1 | KT875642.1 | KT875534.1 | KT875588.1 | KT875480.1 |
| *R. toxicus* | SA03-24 | KT760432.1 | KT875697.1 | KT875427.1 | KT875643.1 | KT875535.1 | KT875589.1 | KT875481.1 |
| *R. toxicus* | SA03-25 | KT760433.1 | KT875698.1 | KT875428.1 | KT875644.1 | KT875536.1 | KT875590.1 | KT875482.1 |
| *R. toxicus* | SA03-26 | KT760434.1 | KT875699.1 | KT875429.1 | KT875645.1 | KT875537.1 | KT875591.1 | KT875483.1 |
| *R. toxicus* | SA03-27 | KT760435.1 | KT875700.1 | KT875430.1 | KT875646.1 | KT875538.1 | KT875592.1 | KT875484.1 |
| *R. toxicus* | SA03-28 | KT760436.1 | KT875701.1 | KT875431.1 | KT875647.1 | KT875539.1 | KT875593.1 | KT875485.1 |
| *R. toxicus* | SA08-03 | KT760437.1 | KT875702.1 | KT875432.1 | KT875648.1 | KT875540.1 | KT875594.1 | KT875486.1 |
| *R. toxicus* | SA08-07 | KT760438.1 | KT875703.1 | KT875433.1 | KT875649.1 | KT875541.1 | KT875595.1 | KT875487.1 |
| *R. toxicus* | SA08-08 | KT760439.1 | KT875704.1 | KT875434.1 | KT875650.1 | KT875542.1 | KT875596.1 | KT875488.1 |
| *R. toxicus* | SA08-09 | KT760440.1 | KT875705.1 | KT875435.1 | KT875651.1 | KT875543.1 | KT875597.1 | KT875489.1 |
| *R. toxicus* | SA08-11 | KT760441.1 | KT875706.1 | KT875436.1 | KT875652.1 | KT875544.1 | KT875598.1 | KT875490.1 |
| *R. toxicus* | SA08-13 | KT760442.1 | KT875707.1 | KT875437.1 | KT875653.1 | KT875545.1 | KT875599.1 | KT875491.1 |
| *R. toxicus* | SA08-16 | KT760443.1 | KT875708.1 | KT875438.1 | KT875654.1 | KT875546.1 | KT875600.1 | KT875492.1 |
| *R. toxicus* | SA19-02 | KT760444.1 | KT875709.1 | KT875439.1 | KT875655.1 | KT875547.1 | KT875601.1 | KT875493.1 |
| *R. toxicus* | SA19-03 | KT760445.1 | KT875710.1 | KT875440.1 | KT875656.1 | KT875548.1 | KT875602.1 | KT875494.1 |
| *R. toxicus* | SA19-04 | KT760446.1 | KT875711.1 | KT875441.1 | KT875657.1 | KT875549.1 | KT875603.1 | KT875495.1 |
| *R. toxicus* | SA19-05 | KT760447.1 | KT875712.1 | KT875442.1 | KT875658.1 | KT875550.1 | KT875604.1 | KT875496.1 |
| *R. toxicus* | SA19-06 | KT760448.1 | KT875713.1 | KT875443.1 | KT875659.1 | KT875551.1 | KT875605.1 | KT875497.1 |
| *R. toxicus* | SA19-07 | KT760449.1 | KT875714.1 | KT875444.1 | KT875660.1 | KT875552.1 | KT875606.1 | KT875498.1 |
| *R. toxicus* | SA19-08 | KT760450.1 | KT875715.1 | KT875445.1 | KT875661.1 | KT875553.1 | KT875607.1 | KT875499.1 |
| *R. toxicus* | SA19-09 | KT760451.1 | KT875716.1 | KT875446.1 | KT875662.1 | KT875554.1 | KT875608.1 | KT875500.1 |
| *R. toxicus* | SA19-10 | KT760452.1 | KT875717.1 | KT875447.1 | KT875663.1 | KT875555.1 | KT875609.1 | KT875501.1 |
| *R. toxicus* | SA19-11 | KT760453.1 | KT875718.1 | KT875448.1 | KT875664.1 | KT875556.1 | KT875610.1 | KT875502.1 |
| *R. toxicus* | SA19-12 | KT760454.1 | KT875719.1 | KT875449.1 | KT875665.1 | KT875557.1 | KT875611.1 | KT875503.1 |
| *R. toxicus* | SA19-13 | KT760455.1 | KT875720.1 | KT875450.1 | KT875666.1 | KT875558.1 | KT875612.1 | KT875504.1 |
| *R. toxicus* | SA19-14 | KT760456.1 | KT875721.1 | KT875451.1 | KT875667.1 | KT875559.1 | KT875613.1 | KT875505.1 |
| *R. toxicus* | SAC3368 | KT760469.1 | KT875734.1 | KT875464.1 | KT875680.1 | KT875572.1 | KT875626.1 | KT875518.1 |
| *R. toxicus* | SAC3387 | KT760468.1 | KT875733.1 | KT875463.1 | KT875679.1 | KT875571.1 | KT875625.1 | KT875517.1 |
| *R. toxicus* | SAC7056 | KT760470.1 | KT875735.1 | KT875465.1 | KT875681.1 | KT875573.1 | KT875627.1 | KT875519.1 |
| *R. toxicus* | WAC3371 | KT760460.1 | KT875725.1 | KT875455.1 | KT875671.1 | KT875563.1 | KT875617.1 | KT875509.1 |
| *R. toxicus* | WAC3372 | KT760461.1 | KT875726.1 | KT875456.1 | KT875672.1 | KT875564.1 | KT875618.1 | KT875510.1 |
| *R. toxicus* | WAC3373 | KT760462.1 | KT875727.1 | KT875457.1 | KT875673.1 | KT875565.1 | KT875619.1 | KT875511.1 |
| *R. toxicus* | WAC3396 | KT760463.1 | KT875728.1 | KT875458.1 | KT875674.1 | KT875566.1 | KT875620.1 | KT875512.1 |
| *R. toxicus* | *FH100 | KT760471.1 | KT875736.1 | KT875466.1 | KT875682.1 | KT875574.1 | KT875628.1 | KT875520.1 |
| *R. toxicus* | *FH83 | KT760465.1 | KT875730.1 | KT875460.1 | KT875676.1 | KT875568.1 | KT875622.1 | KT875514.1 |
| *R. toxicus* | *FH85 | KT760464.1 | KT875729.1 | KT875459.1 | KT875675.1 | KT875567.1 | KT875621.1 | KT875513.1 |
| *R. toxicus* | *FH147 | KT760466.1 | KT875731.1 | KT875461.1 | KT875677.1 | KT875569.1 | KT875623.1 | KT875515.1 |
| *R. toxicus* | *FH141 | KT760467.1 | KT875732.1 | KT875462.1 | KT875678.1 | KT875570.1 | KT875624.1 | KT875516.1 |
| *R. toxicus* | *FH81 | KT760458.1 | KT875723.1 | KT875453.1 | KT875669.1 | KT875561.1 | KT875615.1 | KT875507.1 |
| *R. toxicus* | *FH138 | KT760457.1 | KT875722.1 | KT875452.1 | KT875668.1 | KT875560.1 | KT875614.1 | KT875506.1 |
| *R. toxicus* | *FH87 | KT760459.1 | KT875724.1 | KT875454.1 | KT875670.1 | KT875562.1 | KT875616.1 | KT875508.1 |
| *R. tritici* | WAC7055 | KT760412.1 | - | - | - | - | - | - |
| *R. tritici* | WAC9601 | KT760413.1 | - | - | - | - | - | - |
| *R. tritici* | WAC9602 | KT760414.1 | - | - | - | - | - | - |
| *R. agropyri* | WAC9620 | KT754158.1 | - | - | - | - | - | - |
| *R. agropyri* | WAC9594 | KT754159.1 | - | - | - | - | - | - |
| *R. iranicus* | ICMP 12831 | KT760408.1 | - | - | - | - | - | - |
| *R. iranicus* | ICMP 13126 | KT760409.1 | - | - | - | - | - | - |
| *R. iranicus* | ICMP 13127 | KT760410.1 | - | - | - | - | - | - |
| *R. iranicus* | ICMP 3496 | KT760411.1 | - | - | - | - | - | - |
| *R. rathayi* | ICMP 2579 | KT760417.1 | - | - | - | - | - | - |
| *R. rathayi* | ICMP 2574 | KT760416.1 | - | - | - | - | - | - |
| *R. rathayi* | *^#^*WAC3369 | KT760415.1 | - | - | - | - | - | - |
| *Clavibacter michiganensis* subsp. *nebraskensis* | NCPPB 2581 | **HE614873 | - | - | - | - | - | - |
| *R. caricis* | VKM Ac-1799 | **NR028756 | - | - | - | - | - | - |
| *R. festucae* | DSM 15932 | **AM410683 | - | - | - | - | - | - |
| *R. festucae* | UCM Ac619 | **NR042574 | - | - | - | - | - | - |

*Only DNA was available to us for this study; **Sequences for these species of genus *Rathayibacter* and *Clavibacter* were retrieved from NCBI GenBank; ^#^This isolate was received from culture collection as *R. toxicus* but we identified as *R. rathayi* based on the 16S ribosomal sequences.
